# Spatial-temporal patterns of malaria incidence in Uganda using HMIS data from 2015 to 2019

**DOI:** 10.1186/s12889-020-10007-w

**Published:** 2020-12-14

**Authors:** Simon P. Kigozi, Ruth N. Kigozi, Catherine M. Sebuguzi, Jorge Cano, Damian Rutazaana, Jimmy Opigo, Teun Bousema, Adoke Yeka, Anne Gasasira, Benn Sartorius, Rachel L. Pullan

**Affiliations:** 1 Department of Disease Control, London School of Hygiene & Tropical Medicine, Keppel Street, London, WC1E 7HT UK; 2 Infectious Diseases Research Collaboration, PO Box 7475, Kampala, Uganda; 3 USAID’s Malaria Action Program for Districts, PO Box 8045, Kampala, Uganda; 4 National Malaria Control Division, Uganda Ministry of Health, Kampala, Uganda; 5 Department of Medical Microbiology, Radboud University, Nijmegen, Netherlands; 6 Department of Disease Control and Environmental Health, College of Health Sciences, School of Public Health, Makerere University, PO Box 7072, Kampala, Uganda; 7 African Leaders Malaria Alliance (ALMA), Kampala, Uganda

**Keywords:** Uganda, Malaria, Incidence, Relative risk, Routine surveillance, HMIS, Seasonality

## Abstract

**Background:**

As global progress to reduce malaria transmission continues, it is increasingly important to track changes in malaria incidence rather than prevalence. Risk estimates for Africa have largely underutilized available health management information systems (HMIS) data to monitor trends. This study uses national HMIS data, together with environmental and geographical data, to assess spatial-temporal patterns of malaria incidence at facility catchment level in Uganda, over a recent 5-year period.

**Methods:**

Data reported by 3446 health facilities in Uganda, between July 2015 and September 2019, was analysed. To assess the geographic accessibility of the health facilities network, AccessMod was employed to determine a three-hour cost-distance catchment around each facility. Using confirmed malaria cases and total catchment population by facility, an ecological Bayesian conditional autoregressive spatial-temporal Poisson model was fitted to generate monthly posterior incidence rate estimates, adjusted for caregiver education, rainfall, land surface temperature, night-time light (an indicator of urbanicity), and vegetation index.

**Results:**

An estimated 38.8 million (95% Credible Interval [CI]: 37.9–40.9) confirmed cases of malaria occurred over the period, with a national mean monthly incidence rate of 20.4 (95% CI: 19.9–21.5) cases per 1000, ranging from 8.9 (95% CI: 8.7–9.4) to 36.6 (95% CI: 35.7–38.5) across the study period. Strong seasonality was observed, with June–July experiencing highest peaks and February–March the lowest peaks. There was also considerable geographic heterogeneity in incidence, with health facility catchment relative risk during peak transmission months ranging from 0 to 50.5 (95% CI: 49.0–50.8) times higher than national average. Both districts and health facility catchments showed significant positive spatial autocorrelation; health facility catchments had global Moran’s I = 0.3 (*p* < 0.001) and districts Moran’s I = 0.4 (*p* < 0.001). Notably, significant clusters of high-risk health facility catchments were concentrated in Acholi, West Nile, Karamoja, and East Central – Busoga regions.

**Conclusion:**

Findings showed clear countrywide spatial-temporal patterns with clustering of malaria risk across districts and health facility catchments within high risk regions, which can facilitate targeting of interventions to those areas at highest risk. Moreover, despite high and perennial transmission, seasonality for malaria incidence highlights the potential for optimal and timely implementation of targeted interventions.

**Supplementary Information:**

The online version contains supplementary material available at 10.1186/s12889-020-10007-w.

## Background

The global burden of malaria has declined since 2000 primarily due to the scale-up of control interventions, including long-lasting insecticidal nets (LLINs), indoor residual spraying with insecticide (IRS), and use of artemisinin-based combination therapy (ACT) [1–3]. Nevertheless, incidence rates in sub-Saharan Africa remained high at an estimated 219 cases per 1000 in 2017–2018 [3]. The incidence estimates used to monitor trends across sub-Saharan Africa are typically generated using parasite prevalence in children 2–10 years fitted in prevalence-to-incidence models [3]. Though informative, the surveys included happen infrequently [4] and may be limited in scale. Derived burden estimates, therefore, cannot adequately support day-to-day monitoring for decision making at national or sub-national levels [5].

National malaria control programmes typically depend on routine health management information systems (HMIS) data to guide programme decisions in control and elimination efforts. With the advent and extended access to web-based health information systems, such as the District Health Information System - version 2 (DHIS-2), timely access to nation-wide HMIS data and quality of these data have been shown to have greatly improved in sub-Saharan Africa [6, 7]. As such, the WHO has reiterated that timely and high-quality HMIS-based burden estimates are achievable, and can be used to inform on-going decision making [8]. Despite this, HMIS remains underutilized, especially for risk mapping, due to concerns over incompleteness and delayed reporting [3, 9, 10]. Whilst HMIS has had, and still needs, improvement, substantial discrepancies between estimates of malaria burden from the current prevalence-to-incidence model approach and HMIS-based reports persist among at least 30 high burden countries [3]. Thus, questions remain as to the reliability of HMIS-based estimates and their corresponding representation of fine-scale spatial distribution of risk to support evidence-based decision making by country-level programme managers.

Small area space-time disease models fitted to routinely reported data have been widely implemented to accurately identify contextually important risk factors and unpack spatial-temporal patterns of infectious diseases, including tuberculosis and malaria [11–15]. These models have the capacity to explain the spatial autocorrelation in disease data, and can provide robust means of understanding ecological connectivity and relationships [16] that are critical for control processes in high malaria or other disease burden countries. Moreover, foci of high malaria risk or burden are pertinent to the principle of strategic information to drive impact under the global high burden to high impact initiative, for effective targeting of interventions [17]. This study therefore, aims to investigate a pragmatic novel small-area space-time approach using a nationwide network of health facilities in estimating malaria incidence from HMIS data, in order to identify areas of high malaria burden and risk across Uganda and assess malaria seasonality.

## Methods

### Summary

In brief, the study applied a Bayesian space-time Poisson regression model to assess the spatiotemporal variability of incidence of confirmed malaria (as reported through the national HMIS) at a fine spatial scale (health facility catchment, 3446 catchments with contiguous neighbours ranging from 0 to 11 (Fig. S16, Additional file 1)) by month (July-2015 to September-2019, 51 months), including primary caregiver education, rainfall, land surface temperature, night-time light, vegetation index, and spatial random effects to account for inherent correlation. To do this, health facility catchment cartographies and demographics were developed using multiple sources as described below.

### Study setting

Uganda was estimated to be the 3rd highest contributor of *Plasmodium falciparum* malaria cases globally in 2018, with incidence rates of > 250 cases per 1000 population at risk within a perennial transmission setting [18]. Located between − 1^0^ and 4^0^ latitudes, it covers a total area of ≈241,500 km^2^ that was divided into 15 non-administrative regions (comprised of between one to 13 districts each) considered to be the malaria endemicity zones under the Uganda Demographic and Health Survey (UDHS) Program by 2018 [19]. Nested within these regions were 128 districts (as they were known in 2018), representing the second administrative level of government.

### Data and population

#### Health management information systems data

In Uganda, all health facilities are required to submit monthly reports from their out-patients department (OPD) registers on all reported diseases to the Department of Health information of the Ministry of Health (MoH). Health facilities are either private-for-profit (PFP) or public comprised of the government owned and private-not-for-profit (PNFP) facilities. HMIS was introduced in 1997 as a paper-based reporting system from each health facility to the Ministry of Health. In 2012, however, a web-based reporting version, the DHIS-2, was implemented with full roll-out across the country in 2013 [20]. In this system, health facility data was either entered directly among high-level facilities or sent as paper reports from lower-level facilities to the districts for entry into the online system.

**Fig. 1:**
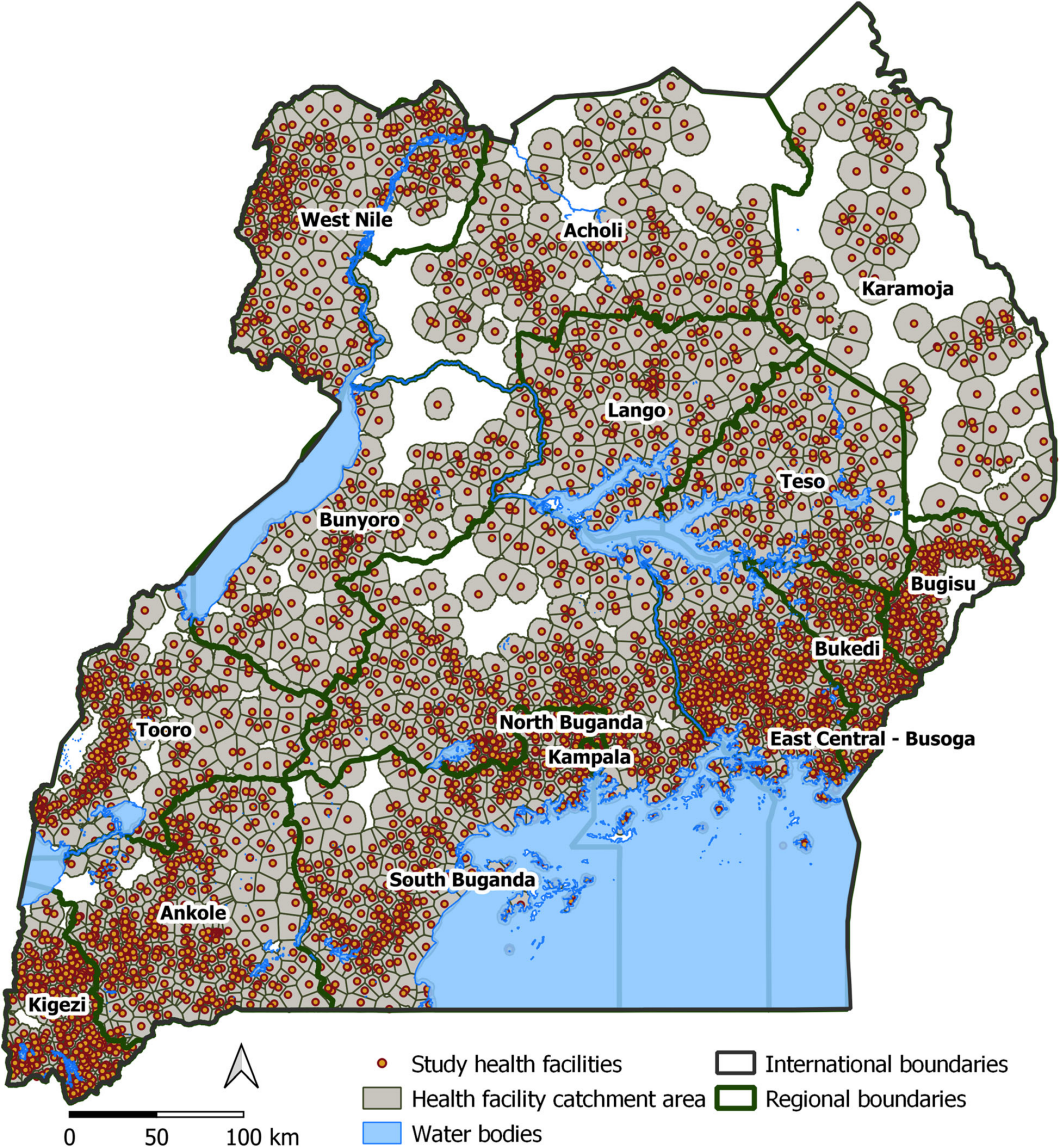
Map of Uganda showing locations of study health facilities within their defined catchment areas. The orange points are the relative geo-locations of the study health facilities recruited from across the country, each situated in a grey background representative of the exclusive catchment area for each facility. The catchment areas were constituted using a three-hour cost distance surface towards each health facility. These are overlaid with the regional boundaries (dark green) defining the 15 endemicity regions across the country. This map was created using open source QGIS 3.12.2 (QGIS.org, 2020. QGIS Geographic Information System. QGIS Association. http://www.qgis.org)

For this study, HMIS data consisted of monthly counts of all reported and confirmed malaria cases from study facilities, defined here as reporting facilities with available geo-coordinates. Reported malaria cases were defined as all cases reported, regardless of confirmation status, while confirmed malaria were laboratory confirmed cases using either blood slide microscopy (B/S) or rapid diagnostic test for malaria (RDT) – per national guidelines. Whereas the recruited reporting facilities with available geo-coordinates represented 3453/7029 (49.1%) of all facilities included within the DHIS-2, 2656/7029 (37.8%) neither reported nor were geolocated and were therefore not recruited (Fig. S1, Additional file 1). Whilst majority of reporting geolocated facilities were publicly owned, the majority of non-geolocated health facilities were private for profit (PFP) commonly located in urban areas and these were excluded. Notably, the two districts of Kampala and Wakiso that together formerly comprised the capital city, contributed 49% of these excluded facilities (Fig. S4, Additional file 1). All reporting facilities that were not geolocated or geolocated facilities without a matching reporting health facility were excluded from this study. A total of 3446 geo-located health facilities constituted the study facilities for this work (Fig. 1).

#### Ancillary data

To define accessibility to health facilities, four categories of single timepoint ancillary data were incorporated to develop a cost-distance surface (Table 1). First, a digital elevation model (DEM) provided a measure of penalty on travel speed depending on direction of travel along the elevation. Second, a land use and land cover raster data set from 2016 was used to define diversity of land cover across which, travel speed would be affected. Third, wetlands, lakes, and rivers were identified as barriers for travel. Lastly, road networks were incorporated and categorized by feasible travel speed class.

**Table 1:** Description of ancillary data sets and the sources of these covariates

Data set	Data type	Data source
**Single time point data sets**		
National geo-located health facilities	Vector	https://figshare.com/articles/Public_health_facilities_in_sub_Saharan_Africa/7725374 Accessed September-2019.
Digital elevation model	Raster	https://www.rcmrd.org/ Accessed October-2019.
Land use and land cover	Raster	http://geoportal.rcmrd.org/layers/servir%3Auganda_sentinel2_lulc2016 Accessed October-2019.
National wetlands	Vector	http://maps.nema.go.ug/layers/geonode%3Augandawetlands2008 Accessed September-2019.
Lakes and rivers	Vector	https://geodata.lib.berkeley.edu/catalog/stanford-fh022bz4757 Accessed September-2019.
Road network	Vector	http://cod.humanitarianresponse.info/sites/default/files/uganda_roads_feb2009.zip Accessed September-2019 and from KEMRI.
**Multi-time point data sets**		
Land surface temperature	Raster	https://earlywarning.usgs.gov/fews/ewx/index.html?region=af Accessed October-2019.
Normalized difference vegetation index (NDVI)	Raster	
Rainfall	Raster	https://www.tamsat.org.uk/data/archive Accessed September-2019.
Night-light emissivity	Raster	https://earthobservatory.nasa.gov/features/NightLights Accessed November-2019.
Mean years of education for women of childbearing age over 2000–2015	Raster	http://ghdx.healthdata.org/record/africa-educational-attainment-geospatial-estimates-2000-2015 Accessed November-2019.

To generate predicted incidence rates, accounting for spatially variable risk factors, ancillary data sets at multi-time points were considered and utilized (Table 1). Notably, whilst vegetation quantities (NDVI) were quantified as the first 10 days (dekad) per month and rainfall as monthly estimates, monthly night-light emissivity was projected using 2012 and 2016 data sets, and the mean number of years of attending school among childbearing women published in [21] were included as a single estimate.

#### Health facility catchments

Currently, the HMIS is used to report malaria burden down to the district level, limiting the ability to observe and act upon heterogeneity at finer spatial scales. In part, this is because of limited information on health facility catchments. Considering proximity as the most important determinant of health facility access and utility [22, 23], health facility catchments were defined based on a cost-distance surface generated using a WHO supported tool known as AccessMod [24] as described in (Section D, Additional file 1). This tool has been widely used in assessments for general and emergency care accessibility, and the estimation of care utilization for febrile illnesses, among others [25–27].

Using the cost-distance surface generated based on anisotropic (direction dependent) analysis, with direction of travel considered as ‘towards the health facility’ in the geographic accessibility model, three-hour travel catchment buffers were generated for each health facility included in the study, given the distribution of travel time (Fig. S5, Additional file 1). To delineate each facility’s catchment area, the intersection polygon between the three-hour travel buffer and a Thiessen polygon around each health facility, generated using ESRI ArcGIS 10.5 *Thiessen polygon* tool (ESRI 1995–2016; Redlands, CA, USA), was derived. This intersection polygon constituted the catchment area for each health facility covering majority of the country.

#### Population data

Population estimates for the country were obtained from gridded population surfaces generated by the WorldPop project whose estimates are based on national census estimates and other factors, accessible from www.worldpop.org. Annual gridded population surfaces were obtained for the duration between 2014 and 2019 and population estimates per year extracted as summary statistics for each calendar year of the study duration 2015 to 2019. These estimates were extracted using ESRI ArcGIS 10.5 *Zonal Statistics* tool at the level of the defined catchment area for each study health facility, regardless of administrative boundaries, given that care seeking is not restricted by these boundaries in Uganda.

### Spatial, temporal, and spatial-temporal analyses

The primary outcome in this analysis was monthly malaria incidence rate, derived from HMIS data as the number of new confirmed cases per facility catchment divided by the total population of the catchment per month (a proxy for person-time).

Inherent spatial correlation of malaria infections is unexplained within classical regression approaches though remains in the residuals and induces spatial autocorrelation in the response even after known available risk factors are accounted for [28]. Using conditional autoregressive models, however, explains this autocorrelation in the outcome using random effects within a Bayesian framework that uses prior distribution, maximum likelihood, and neighbourhood predicts a more reliable outcome [29, 30]. As such, a Bayesian space-time model employing BYM (Besag, York and Mollie) conditional autoregressive random effects and using integrated nested Laplace approximation (INLA) (www.r-inla.org), was fitted to the monthly crude confirmed case rates in R [31]. The model included structured and unstructured spatial effects, as well as structured and unstructured temporal effects, to explain measured (structured) and unmeasured (unstructured) risk factor impacts on the posterior estimates of incidence. The unobserved spatial correlation in the form of noise, attributable to independent health facility catchments, was accounted for through random effects. This model can be summarized as ***=μ + βx***_***i***_ ***+ b***_***i***_ ***+ c***_***i***_, with *y* denoting the posterior estimates of incidence rates, *μ* the crude incidence rates (correlated with the posterior estimates (Fig. S6 and Fig. S7, Additional file 1)), *x*_*i*_ the covariates estimating the risk factors, *b*_*i*_ and *c*_*i*_ the overall spatial and temporal random effects respectively, that are also determined conditional on random effects of neighbouring catchments [32]. Also, to avoid overfitting, a time restriction using a random walk of the first order was included.

Candidate covariates had been used in other studies, given their association with malaria transmission, including rainfall, temperature, vegetation index, night-time lights (a proxy for urbanicity), and caregiver education [13, 33–35]. For inclusion in the final model, covariates quantities were evaluated for impact on a linear regression model of crude incidence rates, considering lower Akaike’s information criteria values (Table S2, Additional file 1). The final covariate list included catchments estimates of mean years of education for women of childbearing age, mean of current and 3 months’ lags for both rainfall and land surface temperature estimates, mean monthly night-time light emissivity, and mean of current and 1 month’s lag of vegetation amounts. All these were significantly associated with crude incidence estimates (Table S3, Additional file 1). Both *β* and *b* were assigned monthly informative Gaussian distributions over the full 51 months length of the study duration. The full model was validated by withholding 20% of data points at random and comparing the model predicted values with the actual observed values using scatter plots and spearman’s correlation coefficients (Section F, Additional file 1).

The relative risk of malaria at district and health facility catchment levels was derived as the respective predicted incidence rate divided by the overall predicted mean incidence rate at the national level per calendar month of the study duration. All maps of the posterior estimates of incidence rates and relative risk of malaria were generated using R (R Foundation for Statistical Computing, Vienna, Austria).

Spatial clustering in the modelled outcome was further investigated using the global Moran’s Index statistic within the spatial dependence (spdep) package of R. This was coupled with visual examination of Moran’s scatter plots of incidence and risk estimates, at both district and health facility catchment resolutions. To identify cluster locations, the local Moran’s Index using ESRI ArcGIS 10.5 *Cluster and Outlier Analysis* (*Anselin Local Moran’s I)* tool was used, set for first order queen contiguity, running 999 permutations and clusters evaluated at 0.01 level of significance.

Also, study model estimates of confirmed malaria cases were compared with estimates from both the WHO’s recent reports [3, 18, 36] and Malaria Atlas Project (MAP) estimates for the same period from https://malariaatlas.org/trends/country/UGA (Section I, Additional file 1) and relationship between MIS regional prevalence estimates [19] and estimated relative risk examined using visual inspection of scatter-plots (Fig. S12, Additional file 1).

## Results

### Study population

The total population identified within the health facility catchments, considered at risk of malaria infection and likely to seek care from the associated geo-located publicly reporting health facility, were considered the study population of interest. The total population was estimated at 34.9 and 39.6 million in 2015 and 2019, respectively, with ≈2.8% of the population located outside of the defined catchments (Section C, Additional file 1).

### HMIS data summary

Between 62.2 and 88.7% of nationally reported cases of malaria annually were diagnostically confirmed cases in 2015 and 2019, respectively (Fig. S2, Additional file 1). Whilst these proportions increased across the 15 regions of the country over time, Kampala recorded marginal improvements. Moreover, the majority of confirmed malaria cases in Kampala (ranging from 61.8 to 81.0% in 2015 and 2018, respectively) were unaccounted for due to exclusion of facilities, leaving only up to 38% of the burden in this metropolitan district estimated (Table S1, Additional file 1). Excluding Kampala, however, results showed that estimates accounted for between 67 to 96% of the routine HMIS-based burden of malaria among the remaining 14 regions, over the study duration. Moreover, in these regions, average annual proportion of reported confirmed cases excluded from the study ranged from 5.3 to 19.8% in Karamoja and Tooro, respectively. Diagnostic testing of suspected malaria cases across the country was conducted either by microscopy or rapid diagnostic tests and reported as a single total.

### Mean incidence rates, seasonality, and risk of malaria

The highest burden regions and districts also hosted health facilities with the highest number of confirmed malaria cases reported. For instance, Bala health centre (HC) III in Kole district of the Lango region reported 3317 cases during November 2015, while Bira HCII in Adjumani district of the West Nile region reported 6697 cases during June 2016. Moreover, Barakala HCIII (highest for two consecutive years) also from West Nile in Yumbe district, reported 9654 cases during October 2017 and 9246 cases during July 2018. Lastly, Matany hospital in Napak district of Karamoja region reported 8089 confirmed cases during September 2019.

**Fig. 2:**
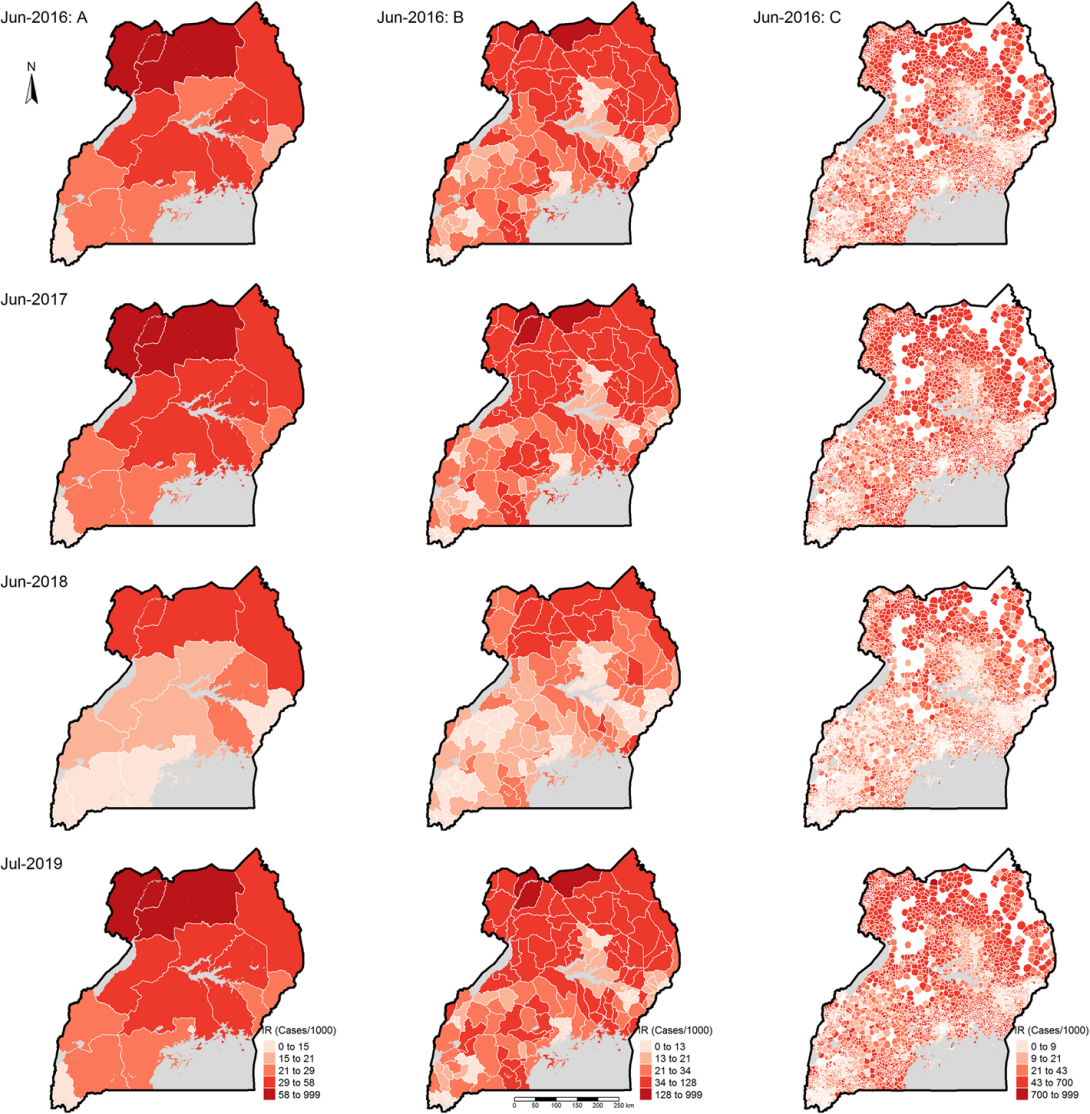
Spatial distribution of malaria incidence rates during high burden months of study duration. Columns A, B, and C represent regions, districts, and heath facility catchments respectively, while the rows correspond to the respective highest burden month of each year. The lighter the shade of colour, the lower the incidence rates within a region, district, or catchment and the darker the colour, the higher the incidence rates

This study showed spatial and temporal variation in incidence rates between regions and districts in any given region, as well as between health facility catchments within districts, both during the low (Fig. S8, Additional file 1) and high burden seasons (Fig. 2).

#### National incidence rates

The model estimated 38.8 (95% CI: 37.9–40.9) million confirmed malaria cases over the study period of July, 2015 to September, 2019, highest in 2016 with 10.3 (95% CI: 9.9–10.7) million cases and lowest in 2018 with 6.5 (95% CI: 6.4–6.9) million cases among complete calendar years (Table S4, Additional file 1). Annual incidence rates reduced from 281.7 (95% CI: 274.9–296.7) in 2016 to 170.0 (95% CI: 165.9–178.8) cases per 1000 in 2018.

**Fig. 3:**
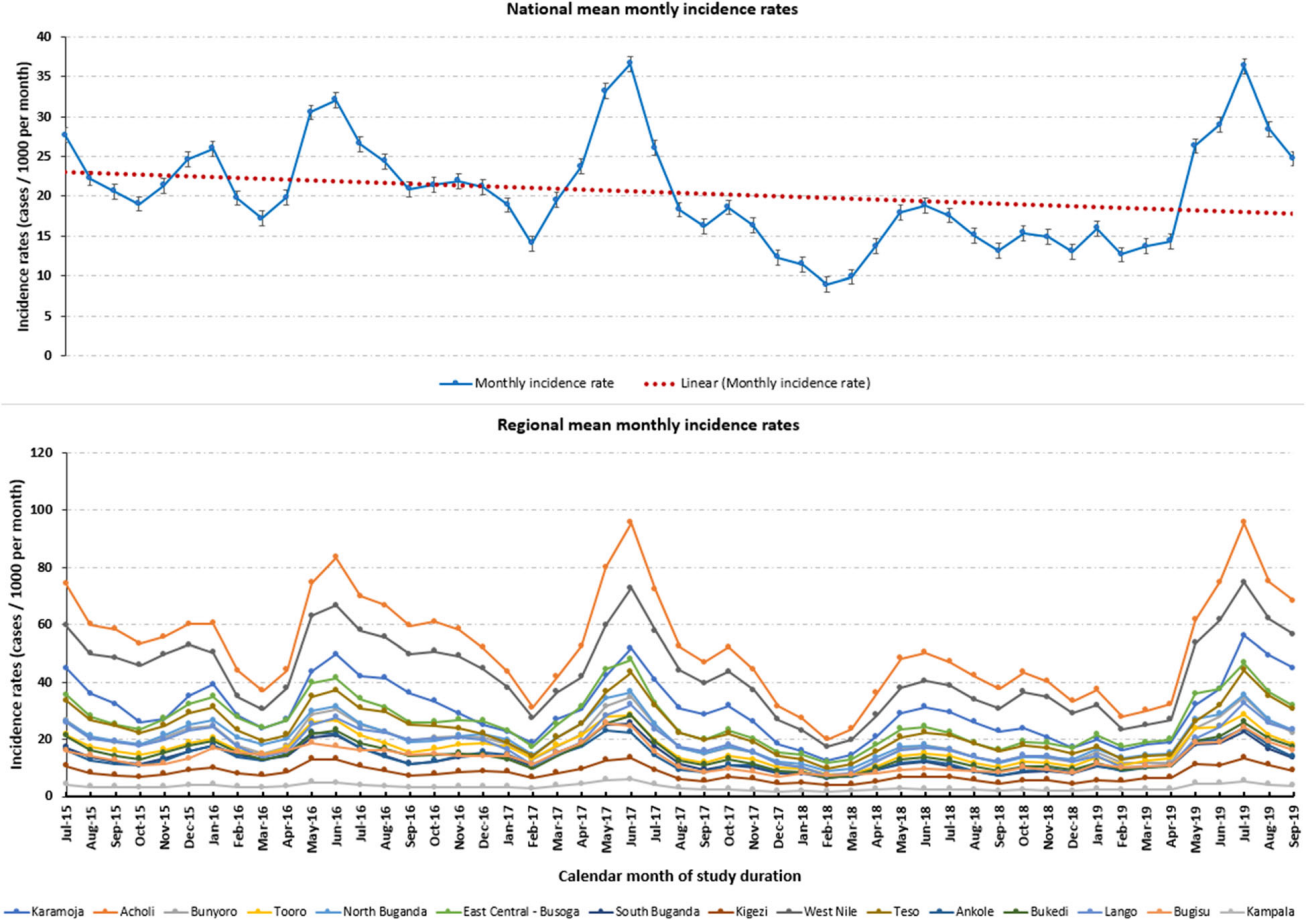
National and regional trends in mean monthly malaria incidence rates July 2015 – September 2019. Trend plots of incidence rates (confirmed malaria cases / 1000) over study time (x-axis) – monthly. The top plot shows the national mean incidence rates per month (blue line) with a linear trend-line (dashed red). The bottom plot shows the trends for the 15 endemicity regions that comprise the country. This Figure was generated using STATA 15 (Stata Corporation, College Station, TX)

Monthly incidence rates showed a general declining trend in the burden of malaria from 2015 to 2019, strongest through 2018 followed by an increase in 2019 (Fig. 3). In all the years of the study, the incidence rates consistently peaked in June and July, reaching a maximum of 36.6 (95% CI: 35.7–38.5) cases per 1000 in June 2017 (Table S5, Additional file 1), at regional (Fig. 3) and district (Fig. S11, Additional file 1) spatial scales. Conversely, low risk periods were less consistent, although often lowest in February and March, reaching a minimum of 8.9 (95% CI: 8.7–9.4) in February 2018.

#### Spatial distribution of incidence rates across the country

Overall, mean monthly regional incidence rates were highest in Acholi region (Northern Uganda) at 52.3 (95% CI: 50.3–59.6) cases per 1000 per month and lowest in Kigezi region (South Western Uganda) at 7.9 (95% CI: 7.6–8.2) cases per 1000 per month (besides Kampala).

Consistent with national trend assessments, monthly trends in regional incidence rates showed the highest peaks in June–July, highest in June, 2017 (Range: 13.4–95.6 cases per 1000) and July, 2019 (Range: 13.5–95.5 cases per 1000 in Kigezi and Acholi, respectively) and the lowest troughs in February–March of each calendar year (Fig. 3). These trends showed that Acholi, West Nile, Karamoja, East Central – Busoga, and Teso persistently recorded the highest monthly incidence rates across the entire study duration. Moreover, the greatest variability in incidence rates was also observed among these five highest burden regions of with respective estimated mean monthly incidence rates of 52.3 (SD: 17.8), 43.3 (13.9), 30.3 (10.4), 26.3 (8.6), and 23.5 (8.0) cases per 1000 per month.

Within these regions, high burden and risk districts were also identified, both during the highest and lowest burden months. During June 2017 district monthly incidence reached the maximum in Lamwo of Acholi, Moyo of West Nile, Kaabong of Karamoja, Namayingo of East Central - Busoga, and Katakwi of Teso regions, at 167.6 (95% CI: 165.6–169.8), 192.5 (95% CI: 189.9–195.1), 81.1 (95% CI: 79.6–82.5), 73.1 (95% CI: 71.9–75.0), 72.0 (95% CI: 70.9–73.1), cases per 1000 per month, respectively (Table S6, Additional file 1).

Monthly incidence rate trends among districts showed that Moyo, Lamwo, Adjumani, Pader, Nwoya, and Maracha persistently recorded the highest monthly incidence rates across the study duration (Fig. 3). Moreover, higher incidence rates were also associated with higher variability in monthly incidence rates with the mean monthly estimate in Moyo at 115.8 (SD: 36.5) and lower rates less variability with Rubanda at 1.6 (SD: 0.5) cases per 1000 (Figs. S10 and S11, Additional file 1).

Within individual districts, a wide distribution of incidence rates was estimated among health facility catchments both during the lowest and highest burden months. From the 3446 catchment areas identified across the country, mean monthly incidence rate reached a maximum of 569.8 (95% CI: 555.2–584.3) cases per 1000 per month in Namayingo district of East Central – Busoga region and minimum of 0.13 (95% CI: 0.10–0.17) cases per 1000 per month in Rukungiri district of Kigezi region, excluding Kampala. Also, higher incidence rates within catchments were associated with higher viability in monthly incidence rates and lower incidence rates with less variability (Fig. S9, Additional file 1). Among health facility catchments, variability in incidence rates reached a maximum standard deviation (SD) = 142.4 cases per 1000 in highest incidence rate catchment located in Namayingo and a minimum SD = 0.1 among the lowest burden catchments in Arua and Kasese districts.

#### Spatial distribution of relative risk across the country

Consistent with incidence rates, relative risk of malaria was highest among the highest burden regions of Acholi, West Nile, Karamoja, East Central – Busoga, and Teso, both during the lowest (Fig. S13, Additional file 1) and highest (Fig. S14, Additional file 1) burden months, maintaining their rank of risk at both times (Table S7, Additional file 1). During the highest burden month of June 2017, the relative risk of malaria among these regions ranged from 1.18 (95% CI: 1.17–1.19) to 2.6 (95% CI: 2.6–2.8)-times higher than national average in Teso and Acholi, respectively. Moreover, while mean relative risk among districts within these regions was higher during the highest burden month at 1.8 (95% Confidence Interval:1.5–2.1) than the lowest at 1.7 (95% Conf. I:1.4–2.0), the difference was not significant (*p* = 0.676) by a two-sample t-test.

**Fig. 4:**
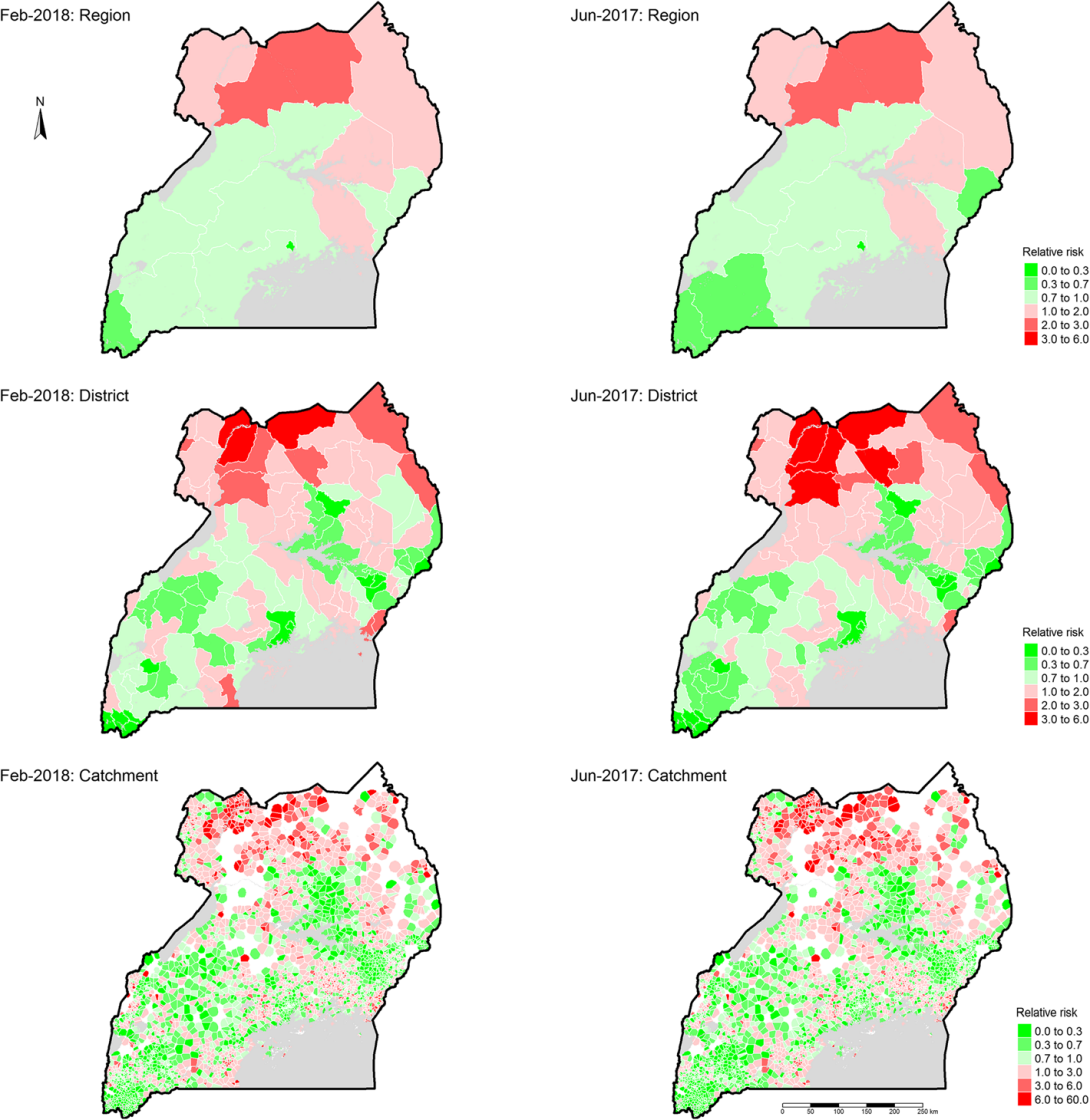
Spatial distribution of the relative risk of malaria during lowest and highest burden months of the study duration. The left column shows, from top to bottom, relative risk by region, district, and health facility catchment for the lowest risk month of February 2018 while the right column shows a similar arrangement for the highest risk month of June 2017. For each row, the same levels (region, district, or health facility catchment) are side-by-side. Green areas are locations with relative risk of malaria lower than the national average where the darker the colour the lower levels of risk below national average. Red coloured areas are locations with relative risk of malaria higher than national average, where the darker the colour the high the risk

Spatial and temporal variation in relative risk observed between regions, and districts within regions (largely informative at programmatic or NMCP levels), was also present between catchments within districts (informative for district health managers). Relative risk remained consistent among the 15 regions, between low and high burden seasons, but showed additional variability among districts and health facility catchments across the two seasons (Fig. 4).

Results showed that catchment risk ranged from 0 to 24.9 (95% CI: 24.4–24.9) times higher than national average during the highest burden month and from 0 to 50.5 (95% CI: 49.0–50.8) during the lowest burden month. Moreover, a non-linear association of catchment risk was observed between the lowest and highest burden months further confirming this rising risk during lower burden months (Fig. S15, Additional file 1). However, the highest risk catchments at the two time points were neither identical nor located in the same district or region.

#### Spatial clustering of risk

Assessment for spatial autocorrelation of incidence and/or risk showed consistent levels of moderate global autocorrelation between both districts (Moran’s I range by month: 0.4 to 0.6, *p* < 0.001) and health facility catchments (0.3 to 0.5, *p* < 0.001). Both during the highest (June-2017) and lowest (February-2018) burden months, global autocorrelation between districts was very similar (Moran’s I = 0.5, *p* < 0.001) (Figs. 17 and 18, Additional file 1) but slight difference between health facility catchments (Moran’s I = 0.4 and 0.3, *p* < 0.001, respectively) (Figs. S19 and S20, Additional file 1).

**Fig. 5:**
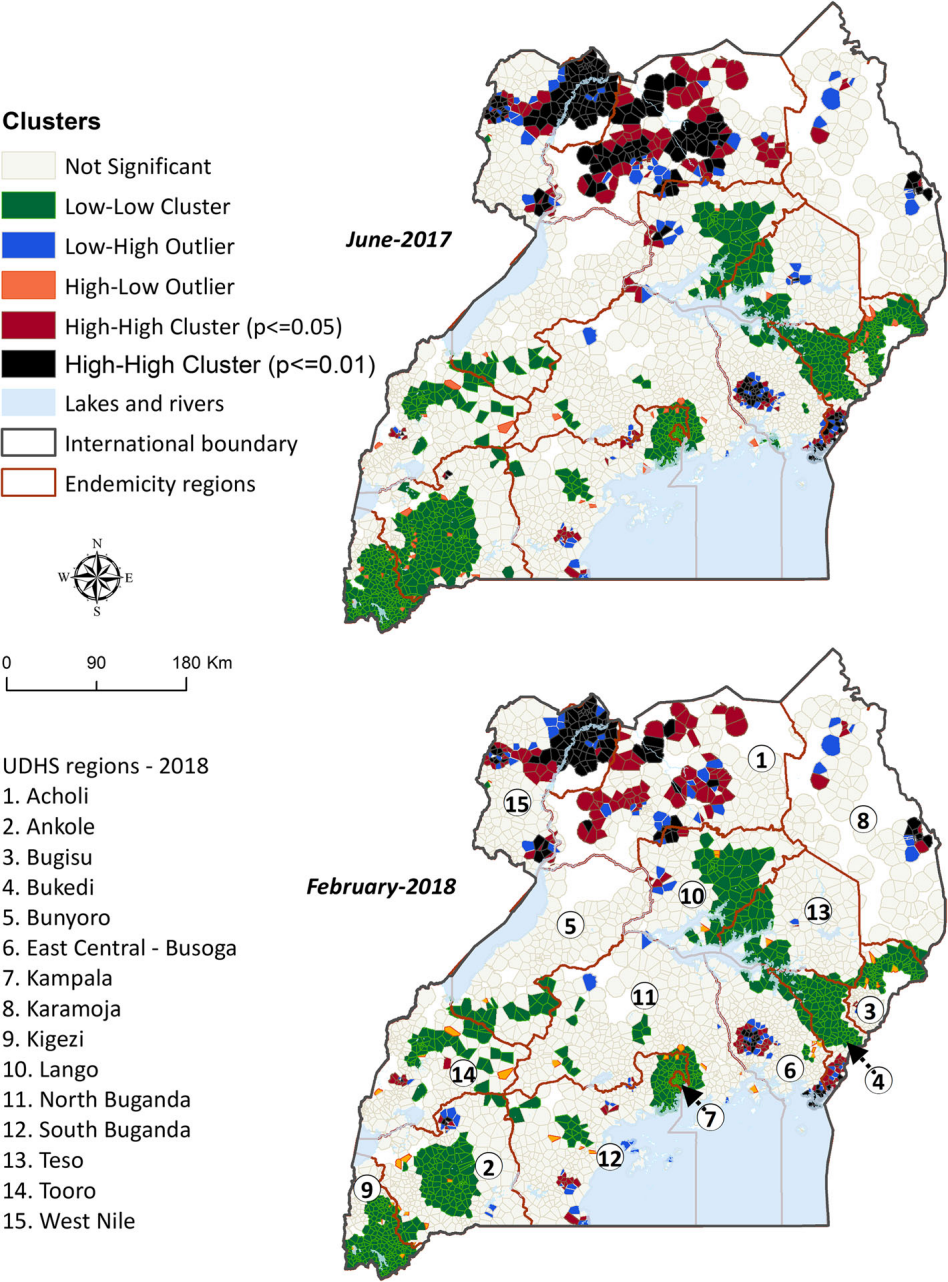
Spatially significant clusters of malaria risk for the highest and lowest burden months between 2015 and 2019, across Uganda. The map at the top represents the distribution of significant clusters of malaria risk across the 15 regions of the country during the highest risk month of June 2017. The map at the bottom represents a similar distribution but for the lowest risk month of February 2018. High-High Clusters: The black and dark red areas represent the clusters of high-risk health facility catchments that are spatially located next to other high-risk catchments, with significant positive spatial autocorrelation at <=0.01 and < =0.05 levels of significance, respectively. This spatial autocorrelation was observed both during the lowest (Fig. S17, Additional file 1) and highest (Fig. S18, Additional file 1) burden seasons. High-Low Outliers: These orange areas represent high-risk clusters that are significantly disparate from their surrounding low-risk catchments. These outliers have significant negative spatial autocorrelation. Low-High Outliers: These blue areas represent the low-risk clusters that are significantly disparate from their surrounding high-risk catchments. These outliers also have significant negative spatial autocorrelation. Low-Low Clusters: These green areas represent the clusters of low-risk health facility catchments that are spatially located next to other low-risk catchments, with significant positive spatial autocorrelation. Not significant: The light grey areas represent the health facility catchments that did not show any significant spatial autocorrelation or clustering of either high, low or outlier distribution of risk of malaria. They are areas of highly random spatial distribution of risk of malaria

Analysis of local spatial autocorrelation at two levels of significance (*p* < =0.05 and *p* < =0.01) identified substantial significant high-high clustering in Acholi and West Nile regions in the North, as well as East Central – Busoga region in the South East of the country, both during the highest and lowest burden seasons (Fig. 5). Similarly, large low-low clustering was identified in the Southern regions of the country. Moreover, outlier catchments typically had significantly lower risk than their neighbours in the north, and higher risk than their neighbours in the rest of the country. Significant monthly high-high clusters were comprised of between 191 health facility catchments during February 2018 and 236 during June 2017 and 2019 (Fig. S21, Additional file 1).

## Discussion

Results from this innovative, large-scale, longitudinal observational study suggest that with improved HMIS reporting, credible high-risk areas at both high and low spatial scales were identifiable. The study revealed a distinct monthly spatial distribution of malaria incidence across the 15 regions of Uganda, in a concurrent multi-resolution assessment, including coarse (regional) down to fine (health facility catchment) spatial resolutions. Moreover, whilst Uganda is considered a perennial transmission setting, this study revealed a nation-wide seasonal pattern in incidence rates with two peaks (major and minor), the highest during June–July and the minor peak during October. This approach may facilitate efficient implementation and optimization of targeted control activities that can leverage existing health facility systems [37]. It may also improve managers’ understanding of the heterogeneity and/or clustering of malaria burden within districts that currently form the lowest level of malaria burden assessments, though acknowledged as difficult to use or unusable for control planning [5].

This study showed that the risk of malaria by regional rank, among the highest and lowest risk regions, had minimal temporal variability. These regions maintained their status both during low and high burden seasons. These findings were consistent with extant UDHS regional stratification of Uganda where Acholi, West Nile, and Karamoja were among the highest transmission regions, and Ankole and Kigezi among the lowest. This stratification supports tailored approaches for long-term malaria control efforts aiming at elimination, as advocated in the global ‘high burden to high impact’ initiative [17] that was recently adopted as central to onward national malaria control strategies for Uganda [38]. Whilst targeted interventions, including IRS [39] and larval source management [40] have been used, further emphasis is necessary [17, 41] with implementation taking greater account of local context. Importantly, however, temporal variability of risk among many regions highlights the continued vital role of routine surveillance for planning and timely action towards control. Moreover, higher risk among high burden locations during the lowest than highest burden seasons suggests persistent high-risk in these locations, the identification of which could facilitate high precision targeted actions for effective control.

This study also identified several distinct clusters of high-risk health facility catchments, which were consistent over time though largest during the highest burden seasons and smallest at the lowest. The largest high-risk clusters were concentrated in the West Nile and Acholi regions in Northern Uganda, although smaller clusters were noted in the recognised high transmission regions of Karamoja and East-Central Busoga [19]. Conversely, the most notable low-risk health facility catchment clusters could be grouped into three categories: highland regions (e.g. Kigezi, Ankole and Bugisu) [42, 43]; regions with recent intense targeted multi-year IRS activity associated with high impacts on transmission (e.g. Bukedi, Teso, and Lango) [4, 19, 44, 45]; and, large urban municipalities (e.g. Southern Buganda) with urbanization associated with reduced transmission [46, 47]. These findings provide further evidence of identifiable candidate locations for targeted control interventions among the high-risk clusters and an approach for assessment of possible impacts of previous interventions.

Trends in annual confirmed malaria cases in Uganda declined between 2016 and 2018, despite increased reporting and proportions of confirmed cases over time, consistent with MIS findings between 2014 and 2018 [4, 19], before a sharp increase in 2019. Moreover, the relationship between regional relative risk and prevalence of malaria (among children under 5 years of age from the 2018 MIS) showed that small changes in parasite prevalence were associated with sharp increases in relative risk among regions at lower than national average risk. However, large changes in parasite prevalence were associated with small changes in relative risk among regions at higher than national average risk. This further confirms the variability of risk among many regions while pointing to strong effects of age on malaria [48]. Besides the estimated confirmed cases being lower than estimates reported by WHO and MAP per year (possibly due to study design of excluding some facilities), trends were also dissimilar with WHO and MAP cases increasing between 2016 and 2017 [18], unlike in this present study. Nevertheless, such dissimilarities have been documented [3] and are likely explained by the use in global assessment for sub-Saharan Africa of prevalence surveys that to date, are predominantly conducted among children [49]. With estimates for the whole population generated from these surveys, despite shifts in malaria burden from children to the older population following effective control interventions [48], the dynamic effects on burden may not be adequately accounted for in the prevalence-to-incidence models used.

The observed seasonality with June–July peaks and February–March troughs was consistent with reports from south western Uganda, where epidemics followed a regular July pattern except during El-nino in 1998 [5, 50] and in Gulu district (Northern Uganda) where between 2006 and 2015 biannual peaks of malaria were reported during June–July and October–November [51]. One study however, reported two peaks of malaria during April–May and September–November in Northern Uganda following the rain seasons, though unsubstantiated [52]. Findings from this present study may inform optimal timing for control activities, including IRS, mass drug administration (MDA), or community mobilization campaigns towards increased malaria risk awareness for control vigilance.

PFP facilities, a small majority of which do not report to the HMIS and were therefore excluded from this study, limit the utility of focal analyses such as presented here. This highlights an important missed surveillance opportunity. The limited capacity to detect outbreaks in settings largely served by PFP may exacerbate the severity of malaria outcomes among their most vulnerable residents, coupled with increased case management costs [53]. There are several possible initiatives to increase reporting in these facilities where a small majority seek care for febrile illnesses [4, 19, 54]. First, provision of guarantees on exclusive use of data for public health not revenue monitoring, may improve confidence and alleviate any fears of punitive intensions in their reporting. Second, ensured availability of standardized reporting tools, may offset running costs of stationery in the private facilities while it enables improved documentation of health records. Third, training of PFP managers and owners on the benefits of surveillance and/or reporting may increase their involvement. Lastly, implementation of regular feedback mechanisms may provide a means of continued evaluation that fosters risk and other assessments that are mutually beneficial.

Given that policymakers’ remediating responses as well as policy formulation processes are informed by pooled information from diverse sources, including but not limited to research, political, and funding provisions, it is unrealistic to expect these technocrats to be expert generators of the evidence from these multi-disciplinary sources. Whilst there are no simple solutions to the implementation of analyses such as in this present study, interpretation of contemporary outputs is nowhere nearly as demanding, highlighting the criticality of partnerships between policy and research dimensions for malaria and other disease control efforts.

This study had limitations. First, the disproportionately low proportion of geolocated reporting private facilities impacted on the estimates of malaria burden, especially among highly urban locations, including Kampala and Wakiso districts and others across the country. Results for the Kampala region (and Wakiso district) in this study, represent only a small proportion of the burden and were excluded from results discussions. Moreover, exclusion of non-geolocated reporting public health facilities (such as in Kitgum district), impacted on the estimates of incidence due to unidentified catchments in those places. Nevertheless, there was wide coverage of health facilities across the country with a small proportion of districts under-represented, minimizing effects of this constraint. Second, the study did not account for level of health facility and other population level factors that impact on differential health seeking behaviour, which may have inflated incidence rates and risk, where a given level or type of facility is preferred. However, in this analysis it was assumed that for uncomplicated malaria, people attend the closest health facility and some important factors such as urbanicity and primary care giver education were accounted for, though further research may be required to better understand impacts of level of health facility on care seeking for uncomplicated malaria. Third, the study did not account for stock levels of antimalarials or test kits, variations of which may impact on the number of cases recorded between seasons of full stock versus stockouts. A better understanding of the linkage between logistics management and HMIS may be required, given known associations between stockouts and increased under-five mortality or compromised treatment practices like dosage rationing and use of less effective remedies [55]. Fourth, given that health facility recruitment into the study was not dynamic, any increase in number of facilities reporting could have had impacts on study findings. Moreover, the systematic exclusion of non-geolocated facilities, may have biased study results towards more long-term established than newer health facilities, but duration of facility existence was beyond the scope of this study.

## Conclusion

Assessment of malaria burden and/or risk in high burden countries using routine surveillance data is highly achievable. Using national routine data, this study provided needed evidence of vital concurrent assessment of malaria risk and burden among regions, districts, and health facility catchments with identifiable significant spatial clustering of risk. Targeting hotspots as an intervention approach has been shown to yield modest and transient impacts on malaria prevalence [56]. However, locations with persistently high-risk of malaria that are potential candidates for health facility-based interventions such as community outreaches, provision of LLINs, mass drug administration and enhanced case management were identified, an approach that may be beneficial beyond isolated health facility catchments. Furthermore, whilst extensive geo-spatial analytical output with scales either too large (region or district) or too fine (pixel or neighbourhood) may be challenging for control programmes to use [57], this study provides evidence of HMIS-based assessments at practical scales for districts to implement and assess intervention impacts. Moreover, in perennial settings, the identifiable strong seasonal patterns as seen with June–July highest peaks and February–March lowest troughs in Uganda, provide vital information for intervention timing. Taken together, these results show the potential in routine HMIS surveillance data for pragmatic timely identification of high-risk areas and with further research, the assessments for optimal implementation of targeted control activities and their impacts.

## Supplementary Information

**Additional file 1.**

## Data Availability

The datasets used and/or analyzed for this study are available from the corresponding author on reasonable request and with permission from the Department of Health Information, Uganda Ministry of Health.

## Abbreviations

ACTs: Artemisinin-based combination therapies
AL: Artemether-lumefantrine
B/S: Blood slide microscopy
BYM: Besag, York and Mollie modelling approach
CI: Credible interval
DEM: Digital elevation model
DHIS-2: District health information system – version 2
DHS: Demographic health survey
HMIS: Health management information system
INLA: Integrated nested Laplace approximation
IRS: Indoor residual spraying with insecticide
KEMRI: Kenya Medical Research Institute
LLIN: Long-lasting insecticidal net
MAP: Malaria Atlas Project
MIS: Malaria indicator survey
MoH: Uganda Ministry of Health
NDVI: Normalized differences vegetation index
NMCP: National malaria control programme
OPD: Outpatient department
PFP: Private for profit
PNFP: Private not for profit
RDT: Rapid diagnostic test for malaria
UBOS: Uganda bureau of statistics
UTM: Universal Transverse Mercator coordinate system
WGS: World Geodetic System of modelling earth
WHO: World Health Organization
