# Supplementary material for: Spatial-temporal patterns of malaria incidence in Uganda using HMIS data from 2015 to 2019
Source: BMC Public Health. 2020 Dec 14;20:1913. doi: 10.1186/s12889-020-10007-w (PMC7737387; doi:10.1186/s12889-020-10007-w)
Supplement: Supplementary file 1 — Additional file 1. [file 12889_2020_10007_MOESM1_ESM.docx]

**Additional file 1**

1. **Study health facilities’ selection**

Health facilities were selected for inclusion in this study primarily in the basis of presence of geo-ordinates from the publicly available database as well existence in the national routine HMIS data repository (DHIS-2) by 2015. The geo-location database contained nearly half the number of entries as the reported malaria cases DHIS-2 database given that 50.9% were without a match in the geo-location database (Fig. S1).

**Fig S1. Flow-diagram of the recruitment process for the study health facilities**


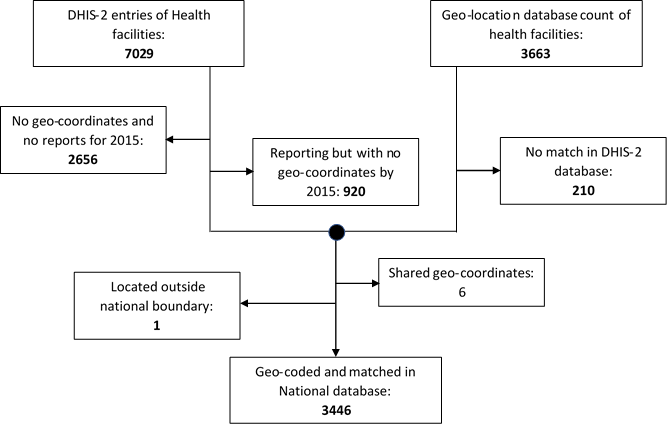


By 2015, there were at least 7029 health facilities (public and private) registered in the DHIS-2 database, in anticipation of them submitting monthly malaria reports. Of these, 2656 (37.8%) did not submit any reports through the year, though majority of these were private clinics that often do not comply with MoH reporting requirements (Fig. S1). A total of 3663 health facilities with associated geo-coordinates were identified by name and these comprised a health facility geolocation database. Comparing the two databases, identities of 3446, by facility name, were matched and the same constituted the study health facilities for this work.

1. **Diagnostic confirmation of malaria and reporting**

Whereas diagnostic testing rates varied across the country, overall proportions of the reported malaria cases confirmed by a diagnostic test increased nationally over time from 62.2 to 88.7% between 2015 and 2019 (Fig. S2). These increases were observed across 14 of the 15 endemicity regions of the country, with the three best performing regions of Lango in Northern Uganda, Kigezi in South Western Uganda and Teso in Eastern Uganda, increasing from 70.9 to 98.1%, 58.8 to 97.4% and 45.8 to 98.1% respectively. Notably however, Kampala in central Uganda recorded declining performance, with the proportion of reported cases that were diagnostically confirmed reducing from 62.0 to 16.0% between 2015 and 2017. Notably however, majority of excluded facilities, were disproportionately concentrated in Kampala (the capital city and most urban district in the country) making it an outlier. Along with the improved diagnostic testing, national reporting rates defined as the proportion of the expected reports received within the DHIS-2 system per year recorded an 22.1% increase between 2015 and 2019 (Fig. S3).

**Fig S2. Distribution of proportions of reported malaria cases that were test confirmed, by the 2018 MIS endemicity regions and nationally.**


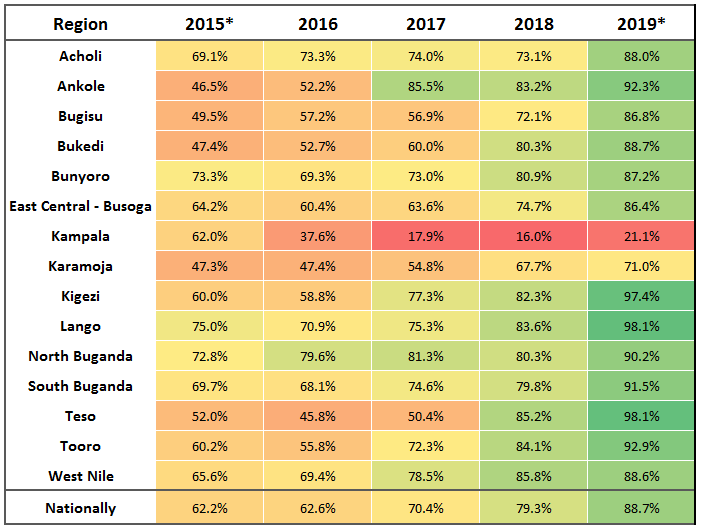


**Not complete calendar years, included July-December 2015 and January-September 2019*

*The red colours represent lowest performance of reporting test confirmed cases while the green colours, improving performance*

1. **Impact of study design on results**

Whilst the study health facilities included in the analysis were only a proportion of the full list of health facilities registered in the national data repository of surveillance data (DHIS-2), these were a good general representation for the national distribution. This was supported by a general left skewed distribution with a median proportion of 71.3% (IQR: 59.0 – 80.0) (Fig. S4) and three districts (Kampala and Wakiso - the most urban settings of the country, plus Kitgum - a fairly rural district in the previously war ravaged Northern Uganda) presenting as outliers with low proportions of registered health facilities included in the analysis (Figs. S3 & S4).

**Fig S3. Distribution of health facility inclusion in the analysis from amongst the list registered in the DHIS-2 database by 2015**


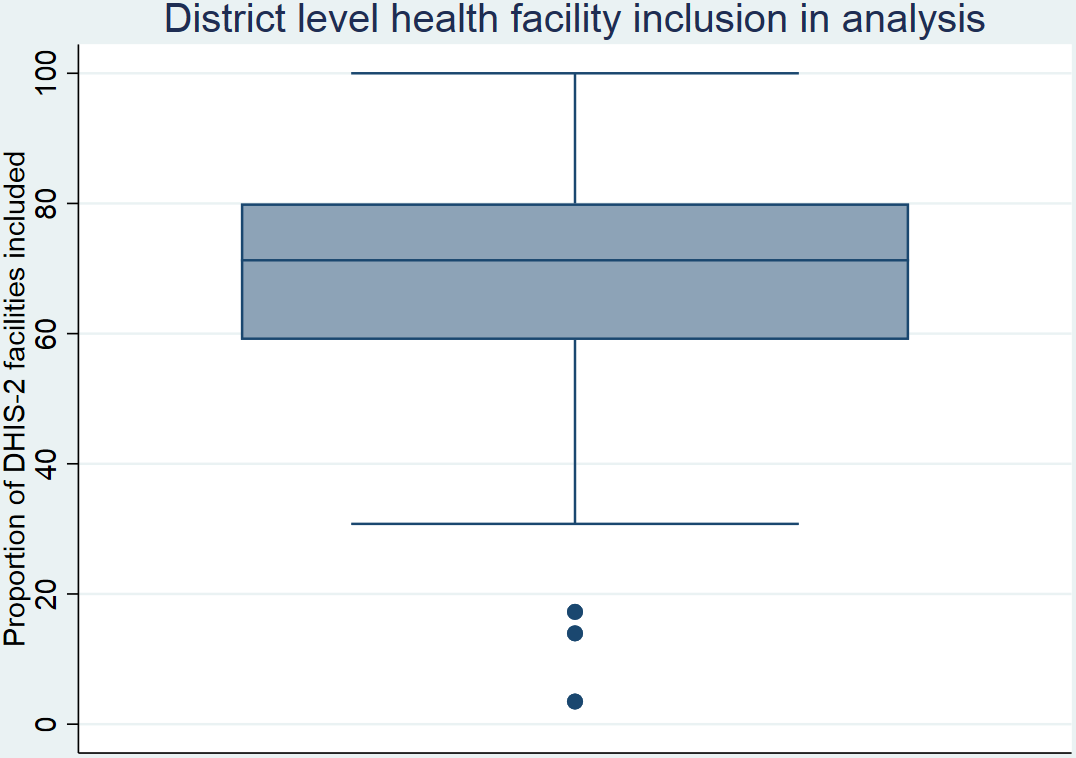


This distribution suggests a fairly good representation of routine surveillance data from across the country with the exception of the three unique districts for which additional efforts would be required to improve their status.

**Fig S4. Geographical representation of the proportion of DHIS-2 health facilities included in this study by district.**


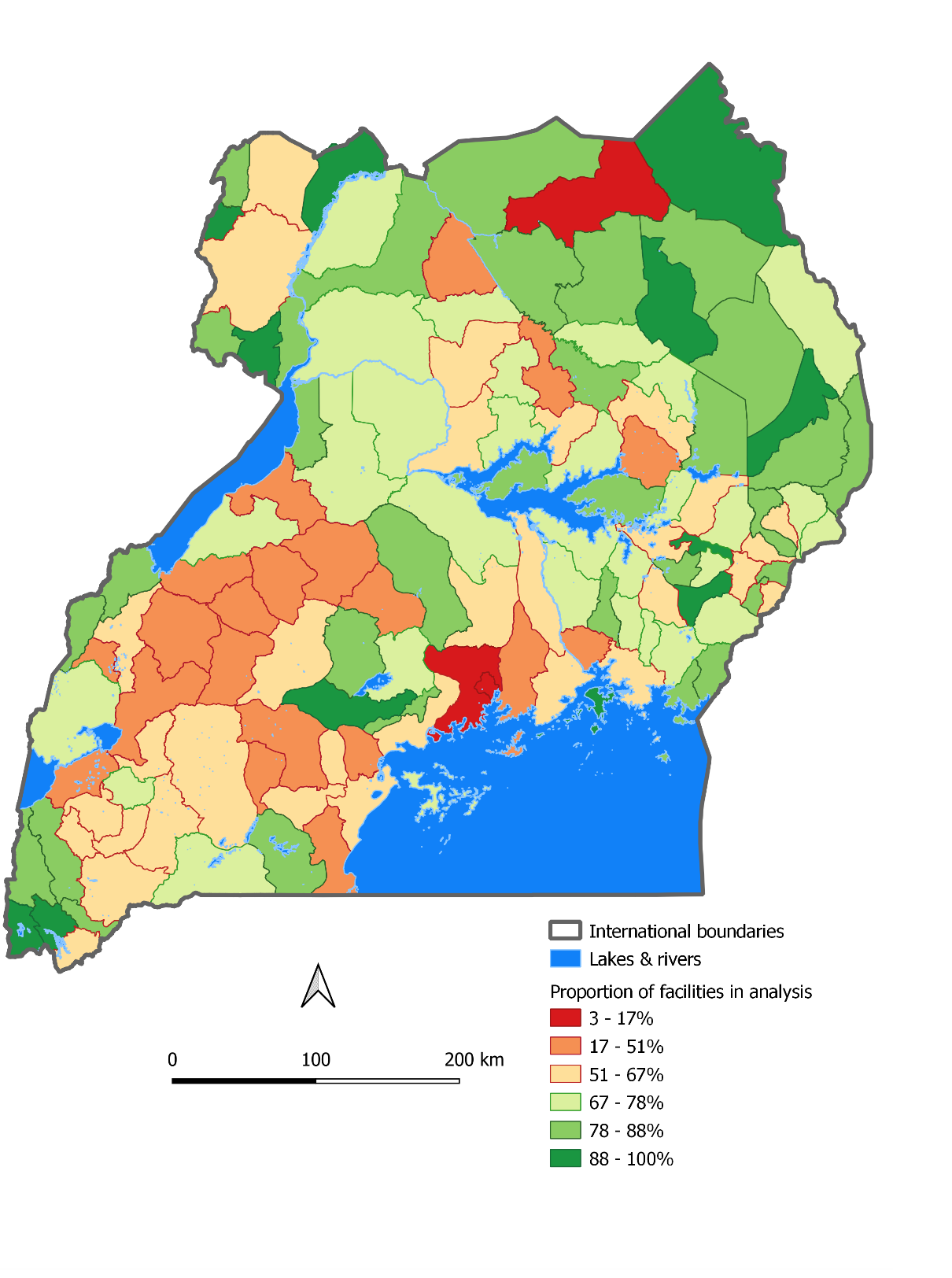


We observed here that the mid-western region of the country is another that is fairly under-represented. However, it’s not clear why in spite of the fairly good overage of catchments across this region, there is still a large number of facilities unaccounted for. Interestingly however, the north-eastern region that had a sparse distribution of catchments than the rest of the country presented some the highest representation districts. Nevertheless, the population in this region leads a characteristically nomadic lifestyle with far-flung permanent settlements among which health facilities would be viable.

To assess the impact of exclusion of facilities from the study on the basis of no geo-location information, however, we examined the raw reported confirmed malaria cases by for each of the 15 endemicity regions. Here, we assessed confirmed malaria cases from excluded regions as a proportion of overall regional confirmed malaria cases reported over each calendar year of the study duration. Results showed that the proportion of confirmed cases among non-geolocated facilities increased slightly between 2015 and 2019 with the highest increase in Tooro from 11.7 to 32.9%, followed by West Nile from 3.6 to 16.4% respectively (Table S1). Overall, the proportion of malaria cases reported from non-study health facilities moderately increased between 2015 and 2019 from 11.7% to 16.1%, which may indicate an increase in reporting, especially from private facilities with time.

**Table S1. Comparison of observed raw (reported) confirmed malaria cases between study and non-study facilities by region, per calendar year**

| **Region** | **2015*** | | **2016** | | **2017** | | **2018** | | **2019*** | |
| --- | --- | --- | --- | --- | --- | --- | --- | --- | --- | --- |
|  | **Included** | **Excluded**  **(% Missed)** | **Included** | **Excluded**  **(% Missed)** | **Included** | **Excluded**  **(% Missed)** | **Included** | **Excluded**  **(% Missed)** | **Included** | Excluded  (% Missed) |
| **Acholi** | 469,343 | 89,535 (16.0%) | 1,011,420 | 230,923 (18.6%) | 648,221 | 158,629 (19.7%) | 405,519 | 92,890 (18.6%) | 1,028,892 | 222,463 (17.8%) |
| **Ankole** | 223,367 | 37,015 (14.2%) | 635,577 | 99,989 (13.6%) | 690,194 | 111,088 (13.9%) | 217,762 | 53,439 (19.7%) | 201,695 | 51,337 (20.3%) |
| **Bugisu** | 121,622 | 15,058 (11.0%) | 299,722 | 35,663 (10.6%) | 316,752 | 44,194 (12.2%) | 199,340 | 35,895 (15.3%) | 242,374 | 42,895 (15.0%) |
| **Bukedi** | 147,273 | 11,500 (7.2%) | 365,374 | 28,915 (7.3%) | 413,057 | 35,863 (8.0%) | 255,022 | 22,221 (8.0%) | 237,194 | 21,658 (8.4%) |
| **Bunyoro** | 190,002 | 27,645 (12.7%) | 485,290 | 79,643 (14.1%) | 496,471 | 70,328 (12.4%) | 298,454 | 48,038 (13.9%) | 460,368 | 67,734 (12.8%) |
| **East Central Busoga** | 459,733 | 58,858 (11.3%) | 1,315,082 | 159,976 (10.8%) | 1,102,382 | 172,844 (13.6%) | 936,740 | 147,221 (13.6%) | 946,492 | 133,679 (12.4%) |
| **Kampala^#^** | 39,321 | 63,481 (61.8%) | 65,791 | 118,693 (64.3%) | 44,852 | 107,817 (70.6%) | 25,245 | 107,912 (81.0%) | 29,196 | 96,983 (76.9%) |
| **Karamoja** | 123,951 | 5,676 (4.4%) | 228,299 | 11,836 (4.9%) | 261,321 | 14,978 (5.4%) | 309,448 | 14,427 (4.5%) | 300,314 | 23,727 (7.3%) |
| **Kigezi** | 63,218 | 4,790 (7.0%) | 151,663 | 19,676 (11.5%) | 160,423 | 21,471 (11.8%) | 80,466 | 11,094 (12.1%) | 94,855 | 7,795 (7.6%) |
| **Lango** | 274,980 | 20,689 (7.0%) | 649,071 | 57,531 (8.1%) | 364,469 | 41,071 (10.1%) | 245,398 | 34,690 (12.4%) | 515,354 | 57,704 (10.1%) |
| **North Buganda** | 359,740 | 37,244 (9.4%) | 899,172 | 104,118 (10.4%) | 1,075,636 | 126,180 (10.5%) | 598,192 | 80,623 (11.9%) | 806,892 | 104,170 (11.4%) |
| **South Buganda** | 339,566 | 50,641 (13.0%) | 857,379 | 132,875 (13.4%) | 924,703 | 180,585 (16.3%) | 446,711 | 124,387 (21.8%) | 511,241 | 114,152 (18.3%) |
| **Teso** | 250,834 | 27,289 (9.8%) | 610,964 | 58,393 (8.7%) | 406,337 | 53,759 (11.7%) | 478,098 | 57,421 (10.7%) | 495,778 | 57,500 (10.4%) |
| **Tooro** | 256,001 | 33,934 (11.7%) | 610,964 | 95,187 (13.5%) | 613,590 | 84,107 (12.1%) | 304,932 | 122,434 (28.7%) | 379,662 | 186,336 (32.9%) |
| **West Nile** | 479,283 | 17,873 (3.6%) | 1,185,521 | 66,045 (5.3%) | 1,367,089 | 132,152 (8.8%) | 1,486,110 | 140,520 (8.6%) | 1,445,541 | 283,897 (16.4%) |
| **Overall** | 3,798,234 | 501,228 (11.7%) | 9,371,289 | 1,299,463 (12.2%) | 8,885,497 | 1,355,066 (13.2%) | 6,287,437 | 1,093,212 (14.8%) | 7,695,848 | 1,472,030 (16.1%) |

******Incomplete calendar years included in study: July-December for 2015 and January – September for 2019*

**^#^** *Region comprised of one district*

Notably, the total predicted number of confirmed cases recorded during the nine months of 2019 included in the study period were higher than the total for all of 2018 by at least 1.4 million cases indicating a major increase in malaria burden and confirming 2018 as the lowest burden year between 2016 and 2019.

**Population estimates:** To determine incidence rates, total confirmed cases at either health facility catchment, district or regional resolution provided the numerator while total population estimates at each respective resolution provided the denominator. Population estimates were extracted from WorldPop gridded surfaces. Compared to Uganda Bureau of Statistics’ (UBOS) national population projection for 2015 of approximately 35.5 million, this study’s estimated Uganda’s at 35.9 million in 2015 from WorldPop. The estimated total study population of 34.9 million therefore, accounted for 97.2% of the 2015 national population estimate. Similarly, whilst UBOS population projection for 2019 was 40.3 million, this study estimated 40.8 million. The study population estimate of 39.6 million during 2019 therefore, accounted for 97.1% of the national population estimate. Whilst the differences between UBOS and WorldPop estimates may be due to in model approaches, differences between national total and study population are attributable to populations located beyond our defined catchments and in locations where very few and sparse health facilities were geolocated.

1. **Cost distance surface**

Generally in Uganda, geographical catchment for each level of health facility have been conceptualized as level II serving a parish, level III a sub-county, and level IV a group of sub-counties otherwise known as a health sub-district (MoH), among others, though it may not be the case in actual practice. Treatment seeking for malaria has been reported as influenced by multiple factors like: knowledge about malaria and its outcomes, severity of disease, reputation of a health facility and affordability of its services, available alternative remedies, as well as age of head of household [1-3]. However, proximity of a health facility may be one of the strongest influences on treatment seeking, often cited in the rampant use of private versus public health facilities [4]. We, therefore, defined health facility catchments under the assumption that people seek care for uncomplicated malaria from the most proximal health facility, using the AccessMod tool supported by the WHO [5].

AccessMod is a web-enabled spatial analysis tool that provides extended ArcView 3.x functionality. Among others, it is used for modelling catchment areas associated with geo-located sources of care as an estimate of physical accessibility, using travel time [5]. Here, first we generated a cost-distance surface of the entire country, at a 100x100 meter pixel resolution. For this, several geographical covariates, within the WGS 1984 UTM Zone 36S coordinate system under the Transverse Mercator projection, were included. Respective covariate classifications were first assigned an intuitive characteristic speed of travel across them, taking into consideration the most likely means of travel useable, to define an overall travel scenario. This scenario included most likely modes of travel across different surfaces such as walking and cycling, driving and riding, as well as a using canoe or boat across water surfaces. Along with these modes, average travel speeds across the respective surfaces or covariates were estimated. The covariates included: 1) a digital elevation model (DEM) of the country that defines the elevation variability across the surface at a 100x100m resolution. This measure of slope per pixel in the DEM was used to penalize the speed of crossing a pixel, particularly for walking and bicycle means, making direction of travel important to define. With interest in access to the health facility in this study, direction of travel was chosen to be towards the health facility. 2) Road network across the country classified as Primary, secondary, tertiary, and other roads, were each considered to enable varied speeds of travel across them, ranging from 30 kilometer per hour (Km/h) on other roads such as feeder or country roads to 100 Km/h on primary roads such as highways. 3) Land use and land cover surface covariate, also at a 100x100 meter resolution, was defined in ten classifications of: tree cover, shrub, grassland, cropland, aquatic vegetation, sparse vegetation or lichens-Mosses, bare ground, built-up, open water, and no data areas. The predominant land cover type per pixel, was assigned a characteristic speed of travel, ranging from a low of one Km/h such as across open water to ten Km/h across bare ground. 4) Lakes and rivers. Whereas the first three covariates were classified to enable travel across them, lakes and rivers were considered primarily as barriers with limited capability to cross them and no likelihood of being residential areas for populations or locations for a health facility. Any health facility that would have its geo-coordinates within water was excluded. This did not include health facilities on islands of which there were several. 5) Another barrier considered in this cost-distance evaluation were swamps with limited likelihood of travel across them except if there was a certain type of road network through them, assumed to likely be a bridge-type crossing. These too were included in this process. Majority of country was within four hours (240 minutes) of travel time to the health facility (Fig. S5) however, there were some outliers, especially far into the lakes and fairly high travel times in parts of the country such as the North-Eastern areas, among others.

**Fig S5. Distribution of travel time to the health facility across the country in minutes from country-wide raster surface generated**


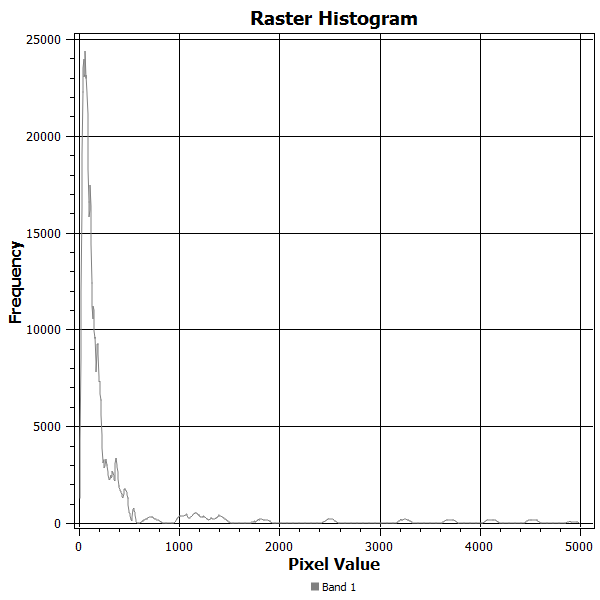


1. **Model selection**

To select the model covariates to include, time varied covariates including rainfall estimates, land surface temperature and vegetation amounts quantified as NDVI were evaluated for selection between the current monthly estimate and the mean of current monthly estimate and either one, two or three months’ lags using akaike’s information criteria values of a multi-variate regression model of crude incidence rates as dependent variable. While keeping all others constant, each covariate was varied to obtain its best quantity for inclusion in the model and the choice of covariate quantity was based on the lowest value of AIC between each covariate’s varied values as summarized in Table S2 below.

**Table S2. Akaike’s information criteria values with corresponding value of covariate included**

| **Covariate** | **Akaike's information criteria (AIC)** | | |
| --- | --- | --- | --- |
|  | **Rainfall** | **Land surface temperature** | **Vegetation (NDVI)** |
| Current month's estimate | -478514.4 | -479,047.50 | -481,472.60 |
| Mean of current & 1 month’s lag | -479,561.30 | -479,232.40 | **-481,537.80** |
| Mean of current & 2 months’ lag | -480,403.10 | -479,743.80 | -481,127.30 |
| Mean of current & 3 months’ lag | **-480,553.30** | **-480,553.30** | -480,553.30 |

*Best choice covariate AIC indicated with bold value*

From the final selection of covariates including: years of education for women of childbearing age, nighttime light emissivity, mean of current and three months’ lags for rainfall and land surface temperature, and mean of current and one month’s lag of NDVI, it was clear that all except years of education for women of childbearing age were significantly and positively associated with the outcome, while education was negatively but also significantly (*p*<0.001) associated as shown in Table S3 below.

**Table S3. Association between best fitted covariates and crude incidence rates from multi-variable regression**

| **Covariate** | **Regression coefficient** | **95% Conf I** | **p-value** |
| --- | --- | --- | --- |
| Education | -0.007338 | (0.007578 - 0.007099) | <0.001 |
| Nighttime light emissivity | 0.000052 | 0.000042 - 0.000063 | <0.001 |
| Mean of current & 1 month’s lag (NDVI) | 0.000477 | 0.000453 - 0.000501 | <0.001 |
| Mean of current & 3 months’ lag (Rainfall) | 0.000128 | 0.000122 - 0.000135 | <0.001 |
| Mean of current & 3 months’ lag (Temperature) | 0.001654 | 0.001584 - 0.001725 | <0.001 |

1. **Bayesian model validation**

The Bayesian model fit to generate the posterior estimates of malaria incidence accounted for four main explanatory factors including: education of women of child-bearing age, the same being the predominant primary care givers in homes; mean rainfall estimates over the current and three previous months; land surface temperature; vegetation amounts, and nighttime emissivity. In order to validate this model, we randomly selected 20% of catchments to be withheld from the posterior estimates and re-run the model to thereafter compared estimates generated from the model with 80% data to posterior estimates determined from the model with the full data.

Results showed that consistent with strong correlation between observed incidence rates and posterior estimates for all data as shown in Fig. S6 below, out of 990 randomly selected catchments, posterior estimates for 942 were within 95% credible interval of the full model prediction for the same. Moreover, there was high correlation between these estimates with Spearman’s rho = 0.6988, P<0.001 also represented in Fig. S7 below.

**Fig S6. Scatter plot of predicted against observed confirmed number of malaria cases from all 3446 catchments in the study**


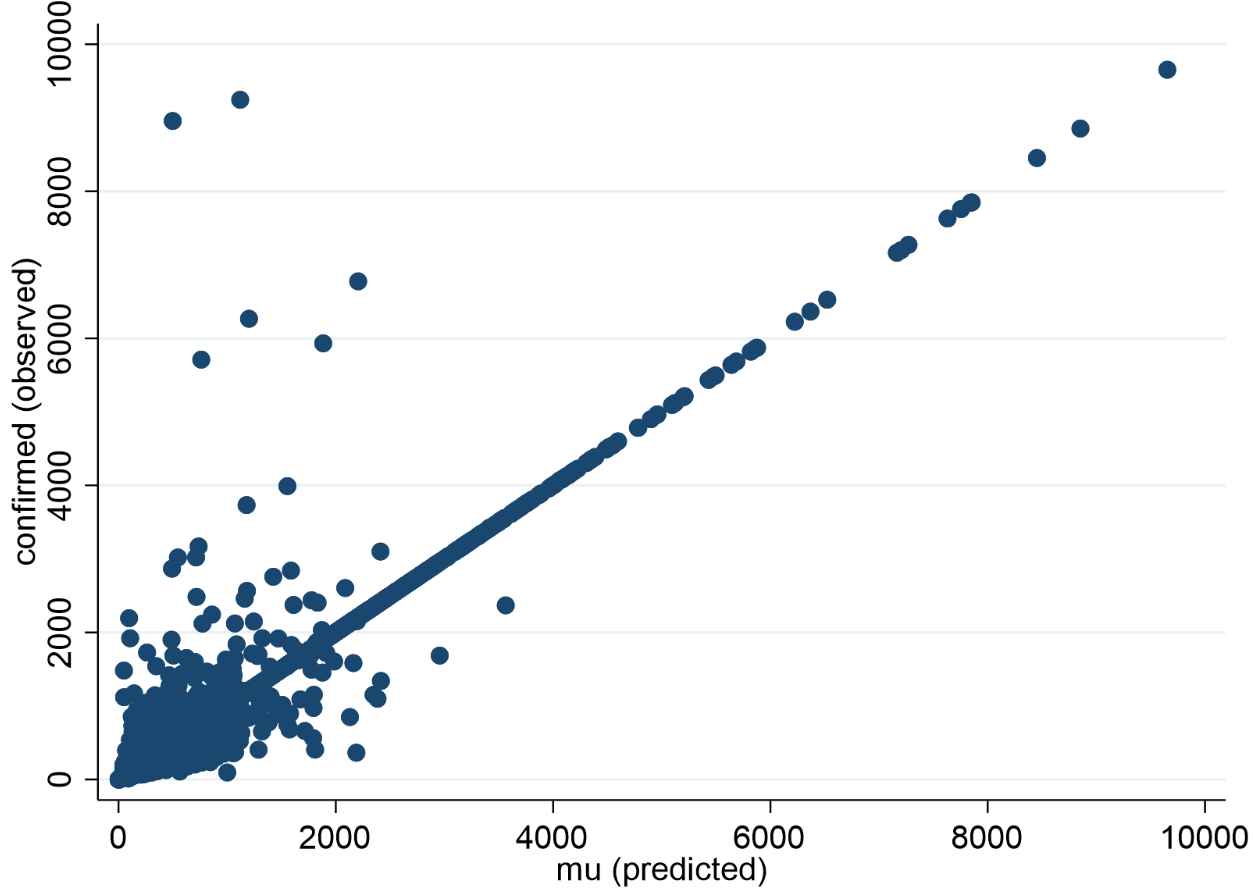


**Fig S7. Scatter plot of model predicted against observed confirmed malaria cases for a random sample of 990 catchments for validation of Bayesian model**


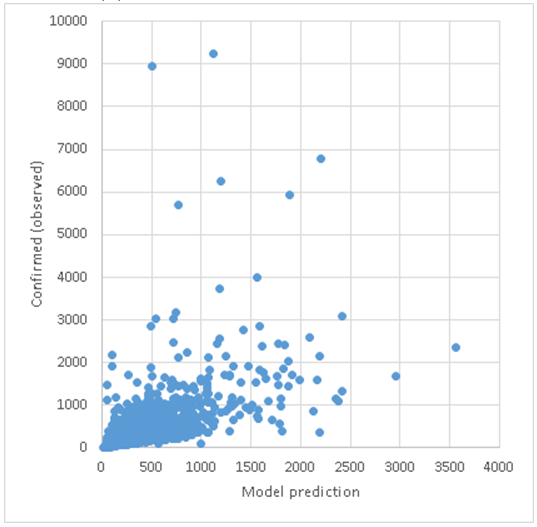


1. **Distribution of catchment-level incidence rates**

Consistent with the observed distribution of incidence rates during the high burden months of the study duration, for all the low burden months, a distinct distribution was observed. In the latter like the former, highest burden districts - potential drivers of their respective regional burden were identifiable. Similarly, highest burden catchments – potential drivers of district burden were also identifiable as shown in Fig. S8 below. Moreover, the pattern of reducing burden from 2016 through 2018, followed by a rebound in 2019 was also observable, particularly among regions and districts.

**Fig S8. Spatial distribution of malaria incidence rates during low burden months of study duration**


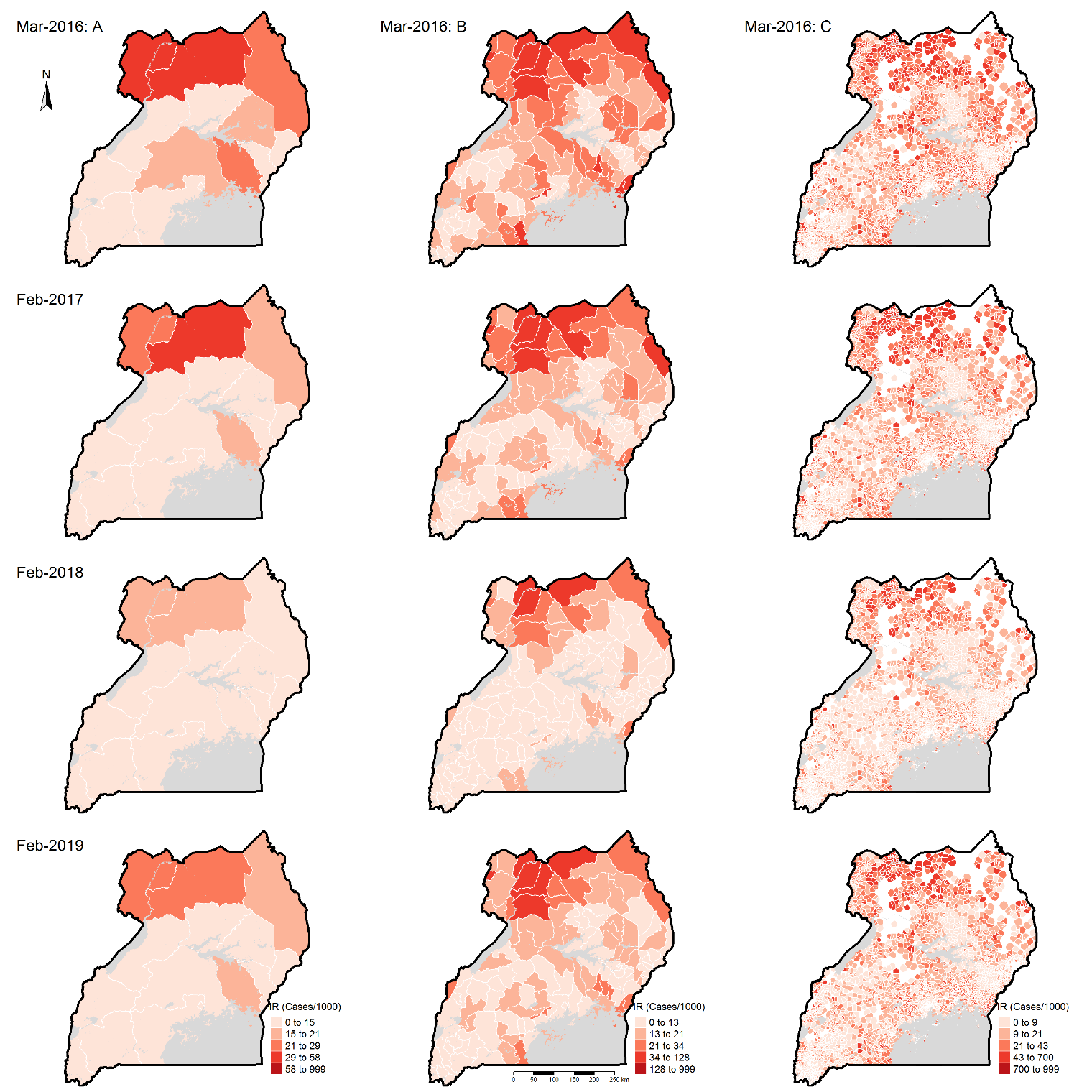


*Maps in column A represent the distribution of incidence rates by MIS regions (15 regions) of the country; B represent the distribution of incidence rates by district (128 districts); while C represent the distribution of incidence rates by health facility catchments (3446 catchments in all).*

Examination of the association between mean and standard deviation (SD) of monthly incidence rates, considering SD the coefficient of variation in these monthly incidence rates, showed that there was a strong linear association between the two, both at district and health facility catchment levels. This was shown using scatter plots of the coefficient of variation versus mean monthly incidence rates at each level as shown in Fig. S9 below as well as the heat-map of district monthly incidence rates in Fig. S10 below. Moreover, increase in mean monthly incidence rates was associated with an increased coefficient of variation and therefore, variability.

**Fig S9. Scatterplot for association between Standard deviation and Mean monthly incidence rates at district and catchment levels**


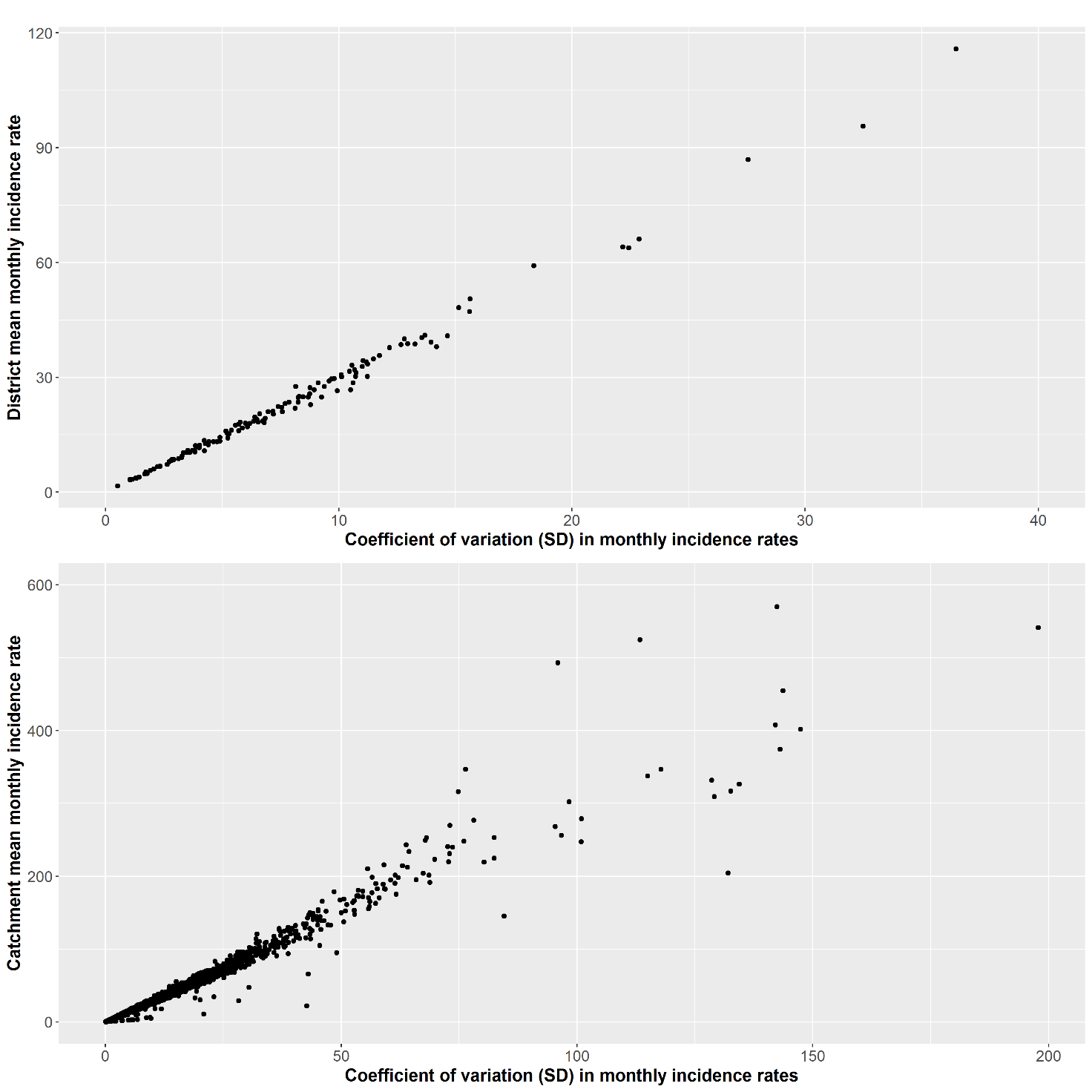


**Fig S10. Distribution of malaria incidence rates per month for the 128 districts in Uganda between July 2015 and September 2019**
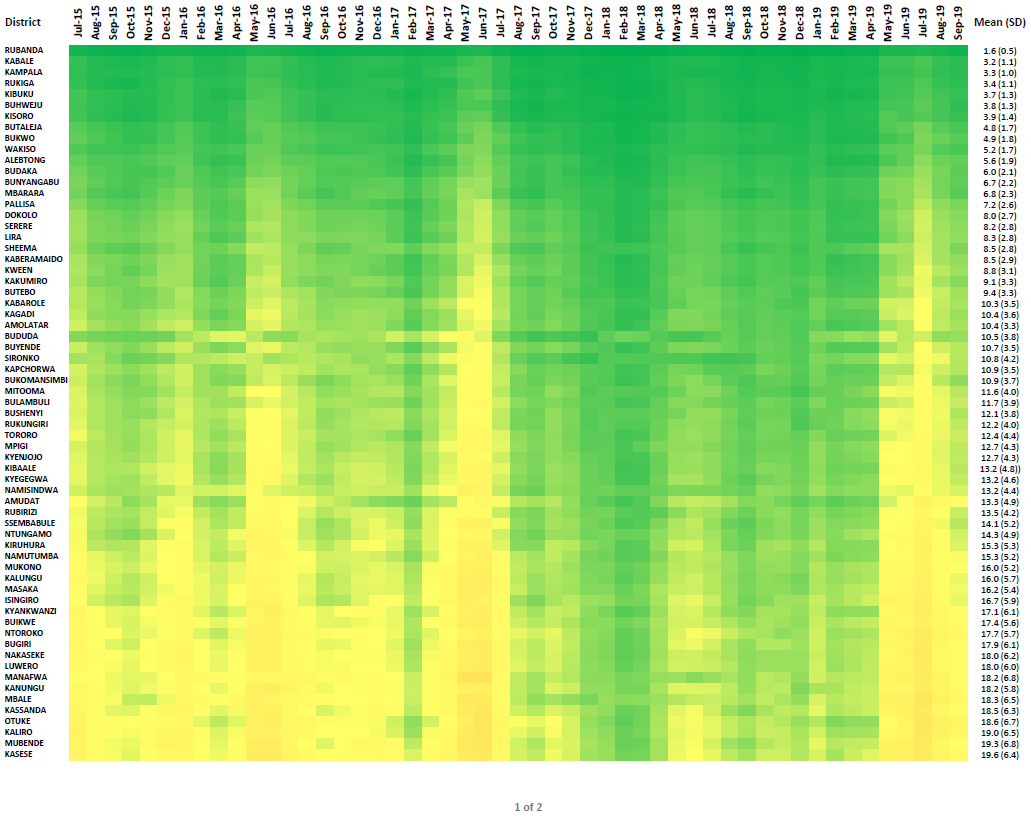


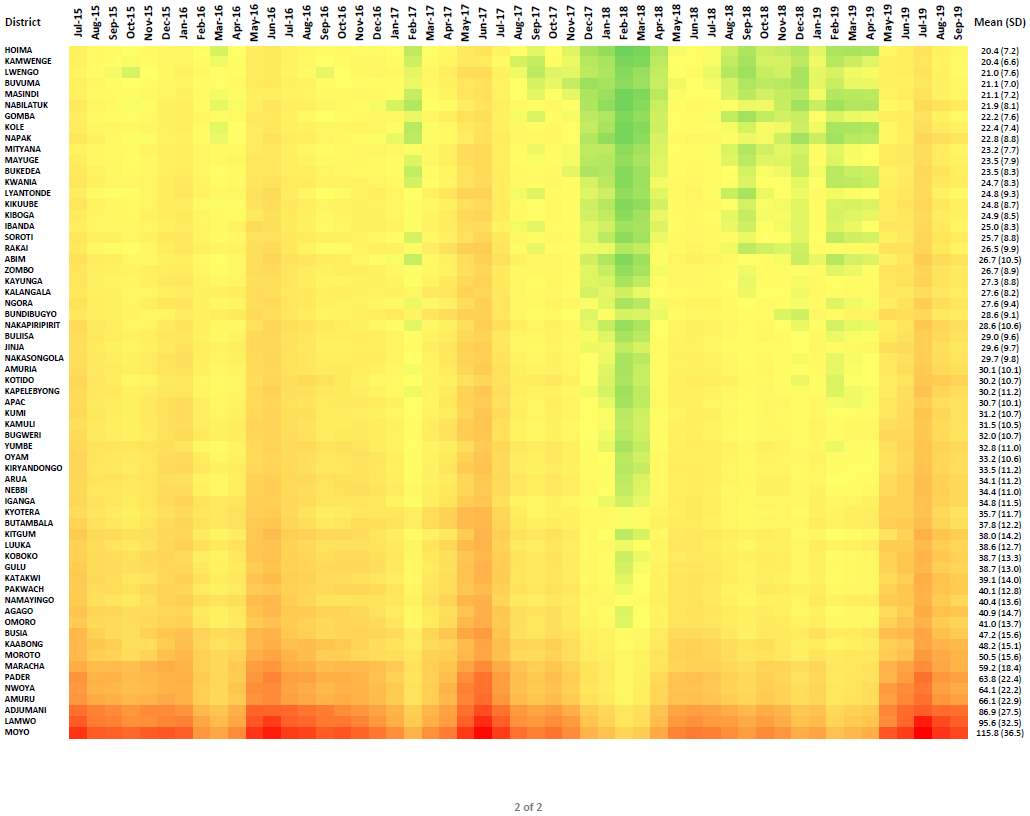


1. **Trends in monthly incidence rates**

Whilst clear and fairly strong trends in malaria incidence rates were observed at the national level with seasonality, similar trends and seasonality were also reflected among regions and districts. The highest burden areas also sustained the highest levels of mean monthly incidence rates across the study duration both at regional and also as shown among districts in Fig. S11 below.

**Fig S11. Trends in the mean monthly incidence rates by the 128 districts of Uganda as of 2018**


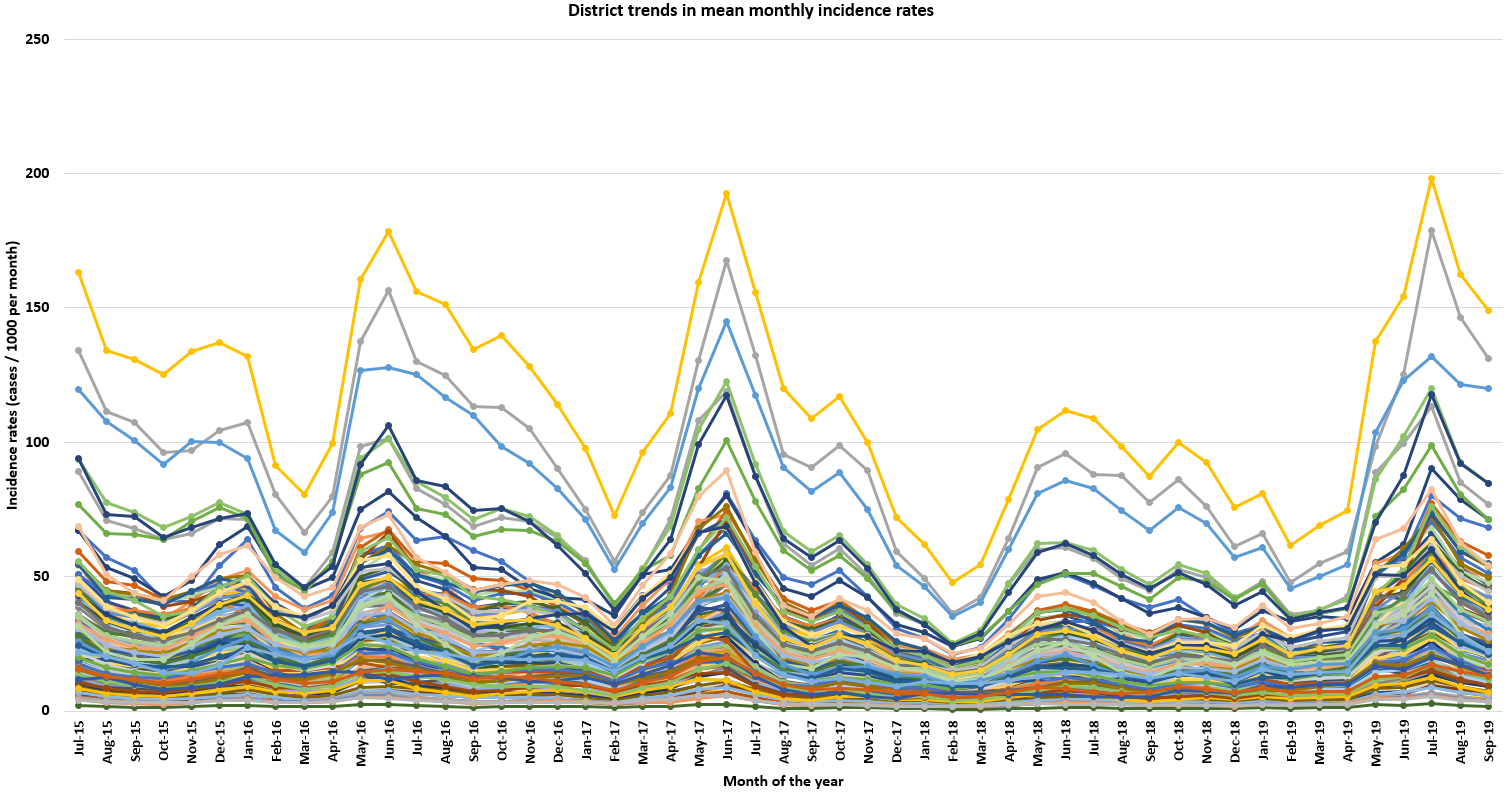


Here, we observe two groups of districts with the highest incidence rates. The first comprised the three districts of Moyo, Lamwo, and Adjumani while the second group included Amuru, Pader, Nwoya, and Maracha. Consistent with seasonality observed at national level, all districts both how and high burden, showed the same seasonality pattern with June-July as the highest incidence rates months and February-March being the lowest incidence rates months across the study duration.

1. **Annual estimates of confirmed malaria cases**

National estimates of annual total confirmed malaria cases in Uganda are obtainable through routine surveillance data. However, these are not the fully utilized in global burden estimates of malaria for Uganda or other countries in the region. To evaluate how HMIS-based estimates compared with global estimates, annual estimates for each calendar year were compared with the most recent estimates reported in the WHO’s world malaria report 2019. Given that global reports are provided in complete years, the respective calendar years for which data included in our study was not from the entire 12 months of the year, the comparisons were considered not applicable. These results are presented in Table S4 below.

**Table S4. Estimated number of confirmed malaria cases from this study compared with estimates from the WHO estimates**

| **Year / duration** | **Confirmed malaria cases in millions (95% CI)** | **Estimated cases from Malaria atlas project in millions (95% Conf. I)** | **WHO malaria report 2019 in millions (95% Conf. I)** |
| --- | --- | --- | --- |
| Jul-Dec, 2015 | 4.759 (4.642 - 5.017) | N/A | N/A |
| Jan-Dec, 2016 | 10.151 (9.904 - 10.688) | 10.876 (8.439 - 14.007) | 12.070 (9.342 - 15.300) |
| Jan-Dec, 2017 | 9.439 (9.210 - 9.927) | 11.096 (8.613 - 14.286) | 13.863 (10.840 - 17.470) |
| Jan-Dec, 2018 | 6.527 (6.368 - 6.865) | N/A | 12.357 (7.623 - 18.970) |
| Jan-Sep, 2019 | 7.951 (7.760 - 8.362) | N/A | N/A |

*CI – Credible interval*

*Conf. I – Confidence interval*

Given that estimates from this study included only the health facilities that were geo-located across the country, we argue that this may explain the lower estimates of confirmed malaria cases per year from this study compared to WHO reported estimates as seen in Table S4. Notably however, the trend observed from this study with total annual confirmed cases reducing between 2016 and 2018 is not observable from WHO reported estimates [6] or with Malaria Atlas project estimates. From this study, 2016 registered the highest total confirmed number of cases and 2018 the lowest of the three years, while the global reports indicated 2017 as the highest with 2016 the lowest of the three years. This may be attributable to differences in the approaches used for the estimates reported between these sources.

**National monthly incidence rates:** Examining monthly estimates of national mean incidence rates, results showed that across the 51 months, June-July experienced the highest incidence rates for all the years, while the lowest estimates were observed variably but mostly during February-March as shown in Table S5. For 2015 where only 7 months were included in this study starting from July, however, the lowest estimate was observed during October.

**Table S5. National, regional and health facility catchment highest and lowest estimated monthly incidence rates per study calendar year in Uganda**

| **Annual duration** | **Peak monthly incidence rates** | | **Lowest monthly incidence rates** | |
| --- | --- | --- | --- | --- |
|  | **month** | **IR (95% CI)** | **month** | **IR (95% CI)** |
| Jul-Dec 2015 | July | 27.6 (27.0 - 29.1) | October | 20.1 (20.6 – 21.7) |
| Jan-Dec 2016 | June | 32.1 (31.3 - 33.8) | March | 17.2 (16.8 - 18.1) |
| Jan-Dec 2017 | June | 36.6 (35.7 - 38.5) | December | 12.3 (12.0 - 13.0) |
| Jan-Dec 2018 | June | 18.9 (18.4 - 19.9) | February | 8.9 (8.7 - 9.4) |
| Jan-Sep 2019 | July | 36.3 (35.4 - 38.1) | February | 12.7 (12.3 - 13.3) |

*CI – Credible interval*

*IR – Incidence rate estimated*

Further, the five highest risk regions both during the highest and lowest burden months being identified across the country as Acholi, West Nile, Karamoja, East Central – Busoga, and Teso. Within these regions, the highest burden districts were also identifiable, the highest four districts in each shown in Table S6 below between the two seasons.

**Table S6. The four highest burden districts within the five highest risk regions across the country during July-2017 and February-2018, the highest and lowest burden months of the study duration.**

|  | **July 2017 (the highest burden month)** | | **February 2018 (the lowest burden month)** | |
| --- | --- | --- | --- | --- |
| **Region** | **District** | **IR (95% CI)** | **District** | **IR (95% CI)** |
| Acholi | Lamwo | 167.6 (165.6 - 169.8) | Lamwo | 36.4 (35.9 - 37.0) |
|  | Amuru | 122.5 (118.4 - 138.0) | Amuru | 25.1 (24.2 - 28.2) |
|  | Nwoya | 118.8 (117.2 - 120.3) | Pader | 24.3 (23.2 - 28.7) |
|  | Pader | 117.3 (111.5 - 118.2) | Nwoya | 23.9 (23.5 - 24.3) |
| West Nile | Moyo | 192.5 (189.9 - 195.1) | Moyo | 48.0 (47.2 - 48.8) |
|  | Adjumani | 145.0 (143.5 - 146.4) | Adjumani | 35.2 (34.8 - 35.7) |
|  | Maracha | 100.8 (99.3 - 102.4) | Maracha | 24.4 (24.1 - 24.8) |
|  | Koboko | 68.5 (67.8 - 69.3) | Pakwach | 17.2 (16.9 - 17.7) |
| Karamoja | Kaabong | 81.1 (79.6 - 82.5) | Moroto | 23.9 (23.4 - 24.4) |
|  | Moroto | 80.2 (78.7 - 81.7) | Kaabong | 21.0 (20.6 - 21.4) |
|  | Nakapiripirit | 52.4 (51.5 - 53.3) | Kotido | 12.6 (12.4 - 12.9) |
|  | Abim | 51.1 (50.1 -52.0) | Nakapiripirit | 11.1 (10.9 - 11.3) |
| East Central - Busoga | Namayingo | 73.1 (71.9 - 75.0) | Namayingo | 19.1 (18.7 - 19.6) |
|  | Luuka | 71.3 (69.9 - 72.7) | Luuka | 17.2 (16.8 - 17.6) |
|  | Iganga | 58.5 (57.6 - 59.5) | Iganga | 15.1 (14.9 - 15.4) |
|  | Bugweri | 58.4 (57.5 - 59.4) | Jinja | 14.1 (13.5 - 16.0) |
| Teso | Katakwi | 72.0 (70.9 - 73.1) | Katakwi | 16.2 (15.9 - 16.5) |
|  | Kumi | 58.6 (57.7 - 59.5) | Kumi | 13.5 (13.3 - 13.7) |
|  | Kapelebyong | 57.5 (56.5 - 58.5) | Ngora | 12.5 (12.3 - 12.7) |
|  | Amuria | 54.2 (53.4 - 55.1) | Amuria | 12.4 (12.1 - 12.6) |

To evaluate the estimated relative risk against other known estimates, a scatter plot of the regional prevalence of malaria estimated among children 0-59 months of age tested by microscopy within the 2018 Malaria Indicator Survey [7], against the study estimated relative risk of malaria during December 2018, the month when the MIS survey was conducted was plotted. Fig. S12 below shows a positive relationship between these two estimates and indication of a positive association between the two.

**Fig S12. Relationship between the 2018 MIS regional prevalence of malaria and estimated relative risk of malaria for December 2018**


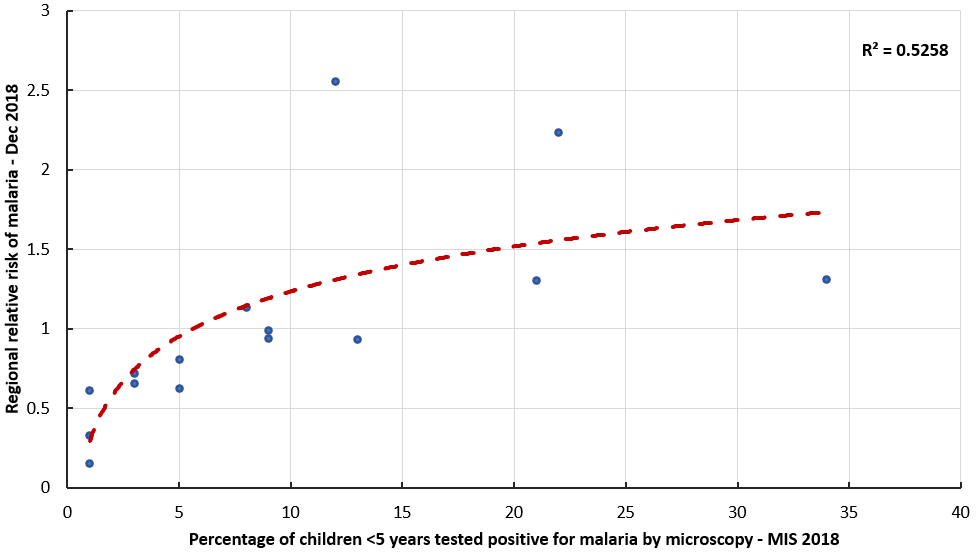


The blue points represent the (prevalence, risk) coordinates and the red dotted line, the fitted curve for the relationship. This observed relationship may provide some evidence of the important effect of age that is largely precluded from evaluations of risk among all populations based on data from children. In these cases, low transmission setting estimates of malaria burden may be under-estimated while being over-estimated in the high transmission settings.

1. **Malaria risk distribution**

Given variability of risk of malaria through the spatial hierarchy, risk at the two higher levels (region and district) was assessed and presented in Table S7 below. The results show regional risk of malaria relative to national average, during the lowest and highest incidence rate months at national level as independent columns as evaluated through national trends in incidence rates. Additionally, for each region, the range of district risk of malaria for the districts that comprise the respective region was also included

**Table S7. Risk of malaria relative to national average, for the lowest and highest burden months between July-2015 and September-2019, by region and district.**

| **Region** | **Lowest burden month**  **(February-2018)** | | **Highest burden month**  **(June-2017)** | |
| --- | --- | --- | --- | --- |
|  | Regional risk | District risk range | Regional risk | District risk range |
| Acholi | 2.2 | 1.5 – 4.1 | 2.6 | 1.9 – 4.6 |
| Ankole | 0.8 | 0.2 – 1.3 | 0.6 | 0.2 – 1.1 |
| Bugisu | 0.8 | 0.2 – 1.1 | 0.7 | 0.3 – 1.0 |
| Bukedi | 0.7 | 1.2 – 2.4 | 0.8 | 0.2 – 2.4 |
| Bunyoro | 0.8 | 0.4 – 1.5 | 0.9 | 0.5 – 1.7 |
| East Central - Busoga | 1.3 | 0.5 – 2.1 | 1.3 | 0.5 – 2 |
| Kampala | 0.2 | N/A | 0.2 | N/A |
| Karamoja | 1.4 | 0.6 – 2.7 | 1.4 | 0.7 – 2.2 |
| Kigezi | 0.4 | 0.1 – 1.0 | 0.4 | 0.1 – 0.8 |
| Lango | 0.8 | 0.3 – 1.5 | 0.9 | 0.3 – 1.6 |
| North Buganda | 1 | 0.7 – 1.4 | 1 | 0.8 – 1.4 |
| South Buganda | 0.7 | 0.3 – 2.0 | 0.7 | 0.3 – 1.9 |
| Teso | 1.1 | 0.4 – 1.8 | 1.2 | 0.4 – 2.0 |
| Tooro | 0.9 | 0.4 – 1.8 | 0.8 | 0.3 – 1.4 |
| West Nile | 1.9 | 1.2 – 5.4 | 2 | 1.3 – 5.3 |

With 15 regions define across the country, we assessed risk distribution by region and the consequent distribution of risk across each region by district. For the low burden month of February-2018, the four highest risk regions in descending order were Acholi, West Nile, Karamoja, and East Central – Busoga, each with greater than one times the national average. Comparatively during the highest burden month of June-2017, the same regions maintained their position in rank of risk (Table S7). On the other hand, the four lowest risk regions during the lowest burden month in ascending order were Kampala, Kigezi, Bukedi, and South Buganda each at lower than national average risk. However, during the highest risk month, the four lowest risk regions changed order to include (in ascending order) Kampala, Kigezi, Ankole and Bugisu, implying that only Kampala and Kigezi maintained their lowest rank of risk. The distribution of risk across districts within each region were further explored using scatter plots for both the lowest and highest burden months as presented in Figs. S13 and S14 below.

**Fig S13**


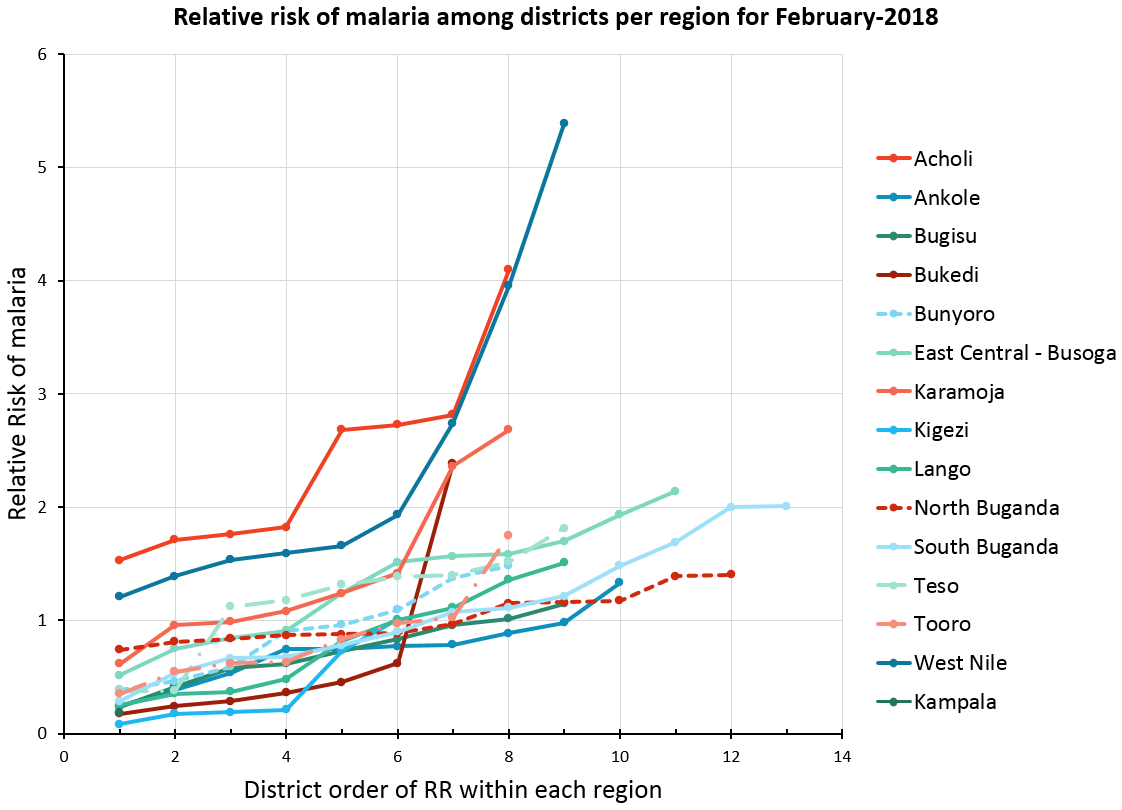


Results showed Acholi and West Nile as the regions with districts that are at the highest risk of malaria, will all their comprising districts at higher relative risk than national average regardless of season. However, while Kigezi region was one of the lowest burden regions with four districts having the lowest risk, the region also had two districts with notably higher risk of malaria than the rest in the region. This pattern is observable among all regions and could play a role in identification of higher priority districts per region over any given observation period.

Notably, among districts within the two lowest risk regions of Kampala and Kigezi the mean relative risk was higher during the lowest burden month at 0.4 (95% Conf. I:0.0 – 0.7) than the highest at 0.3 (95% Conf. I:0.1 – 0.6), but with no significant difference (P=0.706). Similarly, for the middle-ranked risk regions, mean relative risk among their districts was higher during the lowest burden month at 0.9 (95% Conf. I:0.8 – 1.0) than during the highest at 0.8 (95% Conf. I:0.7 – 0.9) with no significant difference (P=0.717).

**Fig S14**


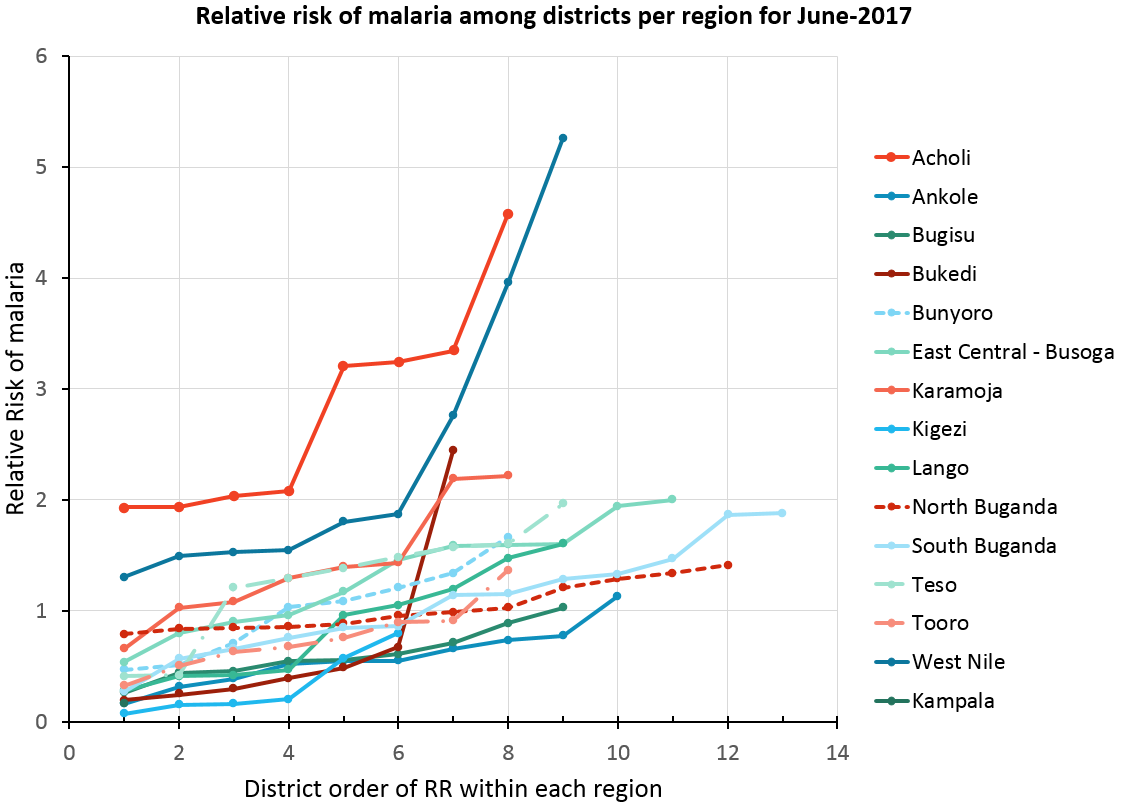


Furthermore, examining the relationship between catchment-level risk during lowest and highest burden months, results showed that the catchments at highest risk were at disproportionately higher risk during lowest than highest burden seasons of the year, as shown in Fig. S15 below.

**Fig S15. Relationship of catchment-level risk of malaria between Lowest and highest burden seasons**


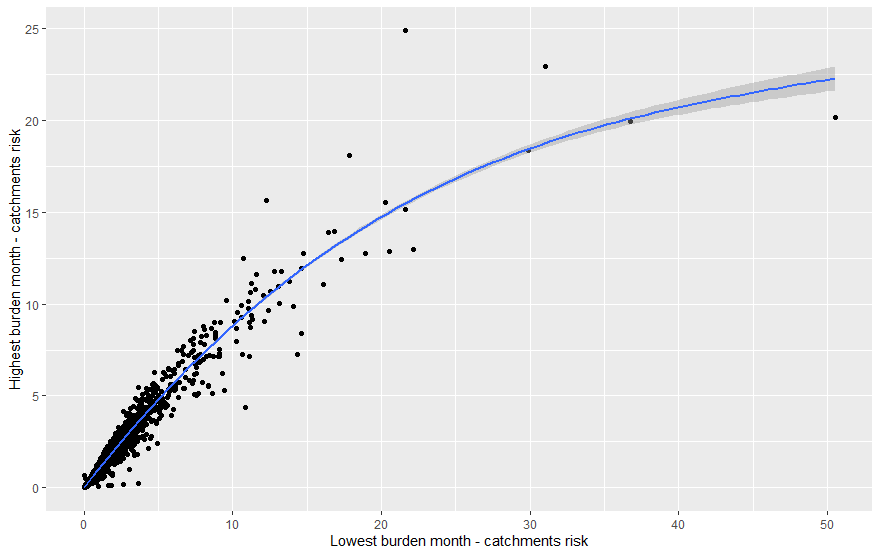


1. **Spatial autocorrelation of risk**

Given the identifiable distribution of risk across the 15 regions of the country, we assessed for spatial autocorrelation of risk at district and catchment levels so as to test for spatial randomness also known as heterogeneity versus spatial clustering of risk of malaria at these scales. For these assessments, the global Moran’s Index (Moran’s I) test was performed in R. At district-level, this included all 128 districts as they were known by 2018. However, owing to the requirement of contiguity among neighbors for this analysis, isolated catchment areas without a neighbor with a shared border were excluded. Consequently, 27 catchments were excluded from this assessment leaving 3419 (99.2%) catchments. Catchment neighborhood was approximately normally distributed as shown in Fig. S16 below, with the highest number of contiguous neighbors a single catchment had being 11 and the least being one.

**Fig S16. Neighborhood distribution among health facility catchment areas.**


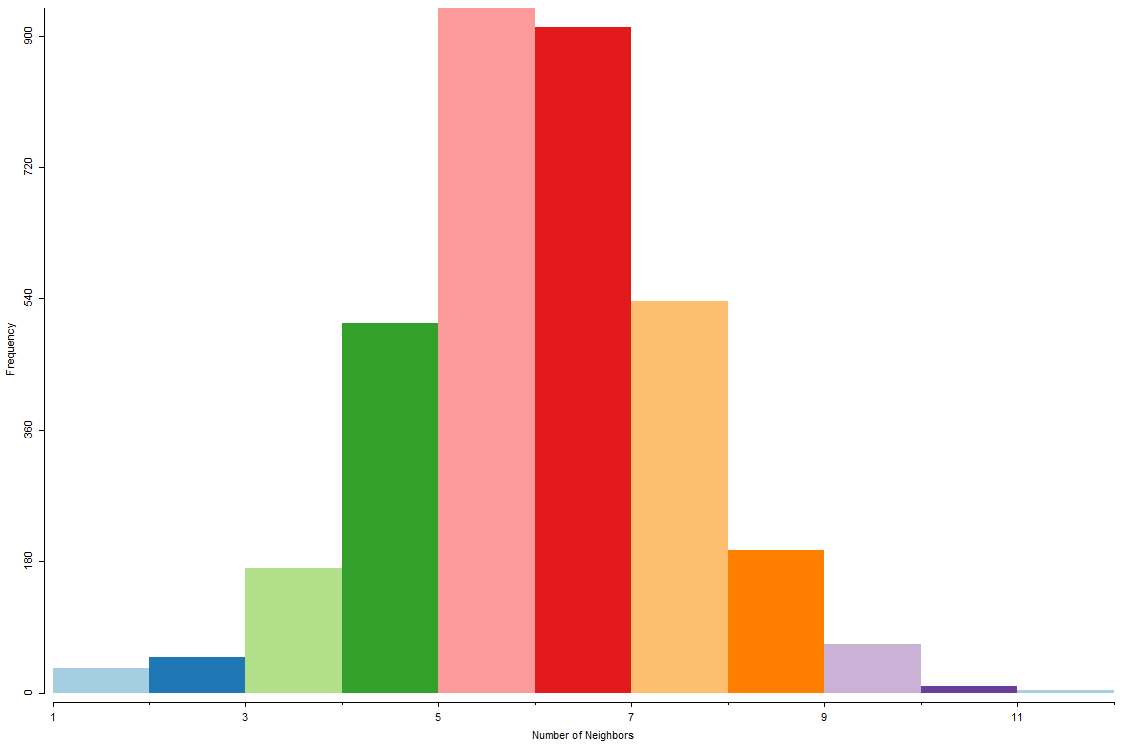


This analysis was conducted for both the lowest and highest burden months of the study duration and results are presented using Moran’s scatter plots for the two durations in Figs. S17 and S18 for the district level and Figs. S19 and S20 at the catchment level below.

The test statistics for spatial autocorrelation (Moran’s I) showed that both at the catchment and district levels, there was increased variability of clustering during the highest burden periods that were associated with increased relative risk. Moreover, we observed higher relative risk among lower transmission areas during lower than higher burden seasons. Together, these may provide some indication of disproportionately higher increase in burden among highest risk locations than increases among the lowest burden areas when malaria upsurges occur. This may be consistent with the notion of the 80:20 Pareto rule [8] indicating here that 20% of the population may bear 80% of the burden of malaria infections.

**Fig S17. Moran's scatter plot for district-level risk of malaria during the lowest burden month of the study duration**


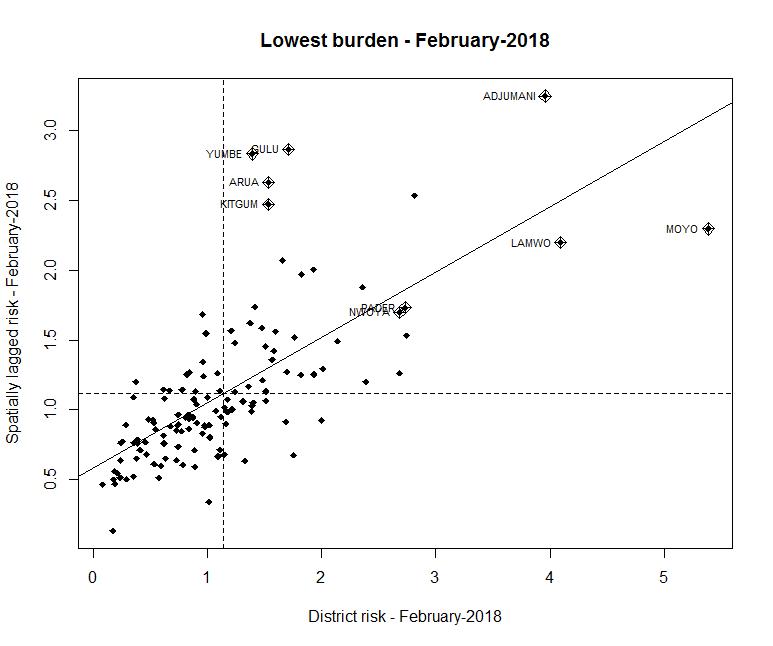


**Fig S18. Moran's scatter plot for district-level risk of malaria during the highest burden month of the study duration**


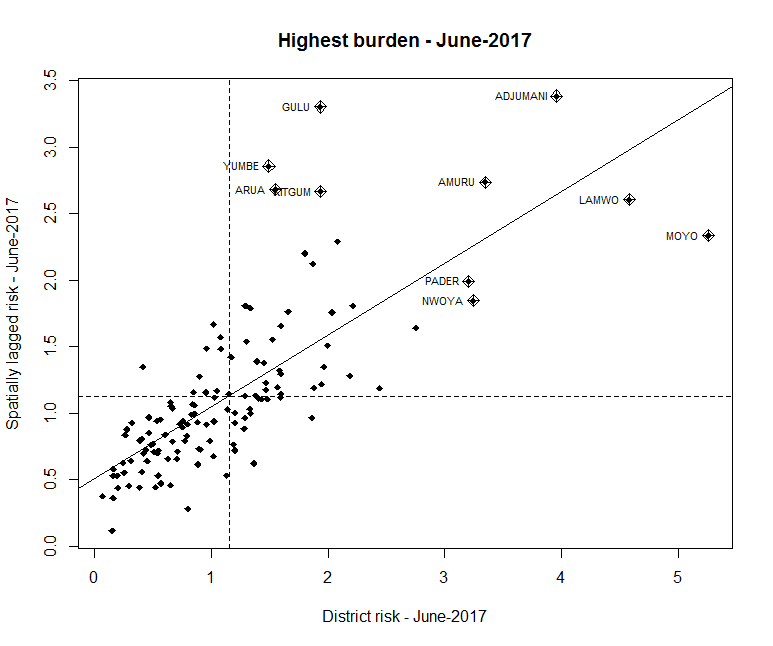


**Fig S19. Moran's scatter plot for catchment-level risk of malaria during the lowest burden month of the study duration**


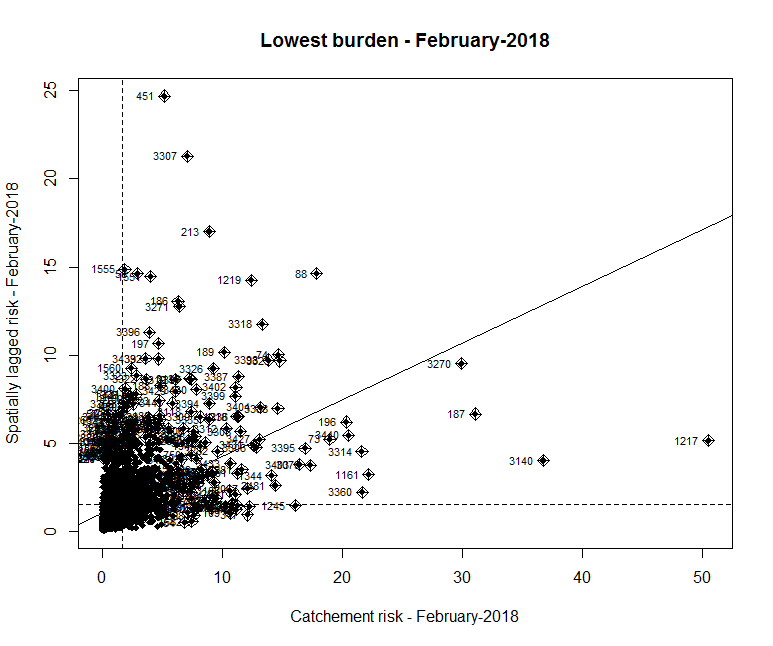


**Fig S20. Moran's scatter plot for catchment-level risk of malaria during the highest burden month of the study duration**


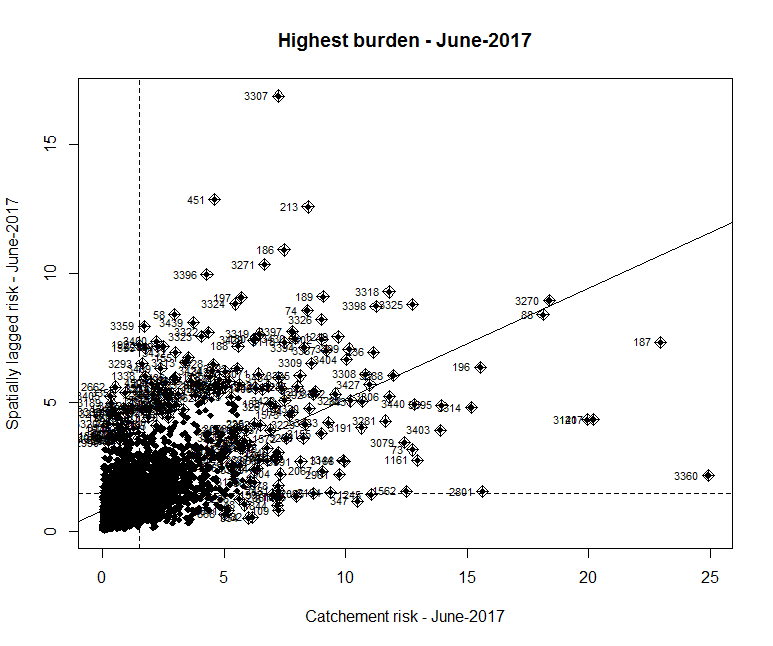


Whilst the location of HH clusters did not change much over time, the number of health facility catchments comprising the identified significant clusters of high-high risk of malaria per month varied over time, showing larger numbers of health facility catchments during high burden seasons that reduced during the low burden seasons (Fig. S21). The total number of health facility catchments was lowest during February 2018 (the lowest burden month) at 191 and highest during both June 2017 and 2019 at 236 health facility catchments.

**Fig S21. Changes in number of catchments comprising monthly high-high clusters of malaria risk, identified using the Local Moran's I statistics**


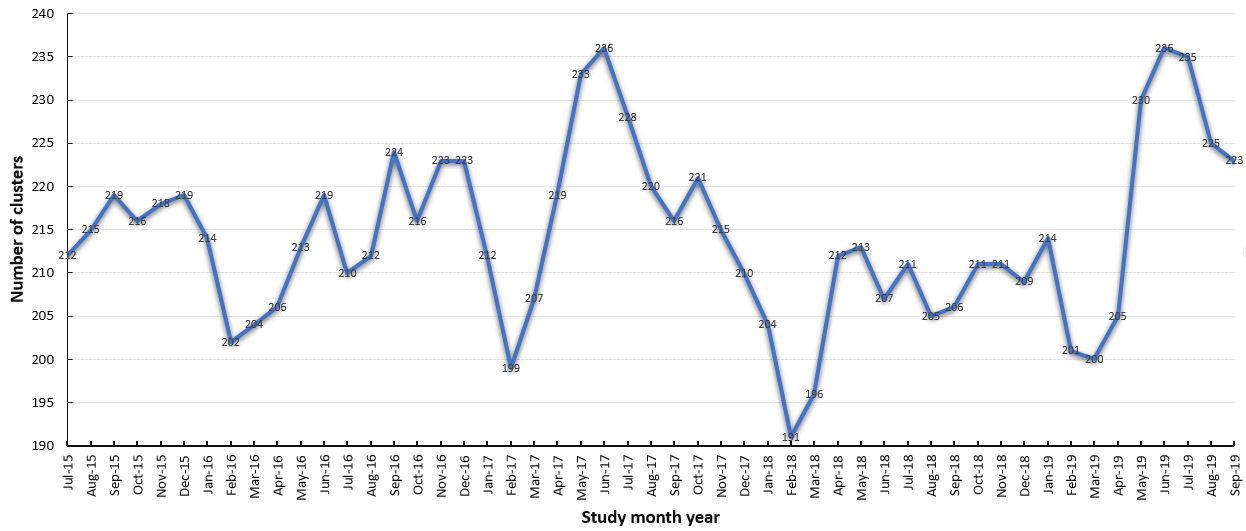


References

1. Amuge, B., et al., *Health-seeking behavior for malaria among child and adult headed households in Rakai district, Uganda.* Afr Health Sci, 2004. **4**(2): p. 119-24.

2. Menon, M.P., J.D. Njau, and D.A. McFarland, *Cost and Predictors of Care-Seeking Behaviors Among Caregivers of Febrile Children-Uganda, 2009.* Am J Trop Med Hyg, 2016. **94**(4): p. 932-7.

3. Tumwesigire, S. and S. Watson, *Health seeking behavior by families of children suspected to have malaria in Kabale: Uganda.* Afr Health Sci, 2002. **2**(3): p. 94-8.

4. Cohen, J., et al., *Determinants of malaria diagnostic uptake in the retail sector: qualitative analysis from focus groups in Uganda.* Malar J, 2015. **14**: p. 89.

5. Ray, N. and S. Ebener, *AccessMod 3.0: computing geographic coverage and accessibility to health care services using anisotropic movement of patients.* Int J Health Geogr, 2008. **7**: p. 63.

6. WHO, *World Malaria Report 2019* 2019, World Health Organization: Geneva, Switzerland.

7. (NMCD), U.N.M.C.D., U.B.o.S. (UBOS), and ICF, *Uganda Malaria Indicator Survey 2018-19*. 2020, NMCD, UBOS, and ICF: Kampala, Uganda, and Rockville, Maryland, USA.

8. Cooper, L., et al., *Pareto rules for malaria super-spreaders and super-spreading.* Nat Commun, 2019. **10**(1): p. 3939.
